# Evaluation of heterologous prime-boost vaccination strategies using chimpanzee adenovirus and modified vaccinia virus for TB subunit vaccination in rhesus macaques

**DOI:** 10.1038/s41541-020-0189-2

**Published:** 2020-05-14

**Authors:** Michel P. M. Vierboom, Agnes L. Chenine, Patricia A. Darrah, Richard A. W. Vervenne, Charelle Boot, Sam O. Hofman, Claudia C. Sombroek, Karin Dijkman, Mohamed A. Khayum, Marieke A. Stammes, Krista G. Haanstra, Chantal Hoffmann, Doris Schmitt, Nathalie Silvestre, Alexander G. White, H. Jacob Borish, Robert A. Seder, Nadia Ouaked, Stephane Leung-Theung-Long, Geneviève Inchauspé, Ravi Anantha, Mary Limbach, Thomas G. Evans, Danilo Casimiro, Maria Lempicki, Dominick J. Laddy, Aurelio Bonavia, Frank A. W. Verreck

**Affiliations:** 1 Department of Parasitology, Biomedical Primate Research Centre, Rijswijk, The Netherlands; 2 Aeras, Rockville, MD 20850 USA; 3 Vaccine Research Center, National Institute of Allergy and Infectious Diseases, National Institutes of Health, Bethesda, MD 20892 USA; 4 Infectious Diseases Department, Transgene SA, ABL Europe Building, Lyon, France; 5 Department of Microbiology and Molecular Genetics, University of Pittsburgh School of Medicine, Pittsburgh, PA USA; 6 GSK, Rixensart, Belgium; 7 International AIDS Vaccine Initiative, New York, NY USA

**Keywords:** Tuberculosis, Adaptive immunity, Infectious diseases, Vaccines

## Abstract

Tuberculosis (TB) still is the principal cause of death from infectious disease and improved vaccination strategies are required to reduce the disease burden and break TB transmission. Here, we investigated different routes of administration of vectored subunit vaccines based on chimpanzee-derived adenovirus serotype-3 (ChAd3) for homologous prime-boosting and modified vaccinia virus Ankara (MVA) for heterologous boosting with both vaccine vectors expressing the same antigens from *Mycobacterium tuberculosis* (Ag85B, ESAT6, Rv2626, Rv1733, RpfD). Prime-boost strategies were evaluated for immunogenicity and protective efficacy in highly susceptible rhesus macaques. A fully parenteral administration regimen was compared to exclusive respiratory mucosal administration, while parenteral ChAd3-5Ag prime-boosting and mucosal MVA-5Ag boosting were applied as a push-and-pull strategy from the periphery to the lung. Immune analyses corroborated compartmentalized responses induced by parenteral versus mucosal vaccination. Despite eliciting TB-specific immune responses, none of the investigational regimes conferred a protective effect by standard readouts of TB compared to non-vaccinated controls, while lack of protection by BCG underpinned the stringency of this non-human primate test modality. Yet, TB manifestation after full parenteral vaccination was significantly less compared to exclusive mucosal vaccination.

## Introduction

Tuberculosis (TB) is one of the top 10 causes of death worldwide and the leading cause of death from a single infectious agent^1^. Control of TB by antibiotics is inefficient and further complicated by the increasing emergence of multidrug-resistant (MDR) and extensively drug-resistant (XDR) *Mycobacterium tuberculosis* (*Mtb*) strains^2,3^. Prophylactic vaccination is the most effective way to break the TB transmission cycle^4,5^. For almost a century, the only available vaccine against TB has been *Mycobacterium bovis*-derived Bacillus Calmette Guérin (BCG), which particularly protects newborns from *Mtb*-associated meningeal and miliary tuberculous disease^6,7^. However, the efficacy of BCG in protecting adolescents and adults from the most common and transmitting form of pulmonary TB is poor and highly variable in different populations^8,9^. A recent revaccination study in South Africa^10^ showed a 45% reduced risk of infection in adolescents by reduction of sustained QuantiFERON conversion as a surrogate readout. Still, conventional BCG vaccination has a limited impact on the TB pandemic and we are in dire need of new, safe, and more effective vaccination strategies.

Amongst the various approaches that are being deployed toward the development of improved TB vaccines is the exploration of viral vectors that are adaptable by genetic engineering to express specific *Mtb* sequences as vaccine targets^11^. While recombinant proteins as subunit vaccines require an immune-potentiating adjuvant formulation, viral vectors intrinsically harbor pathogen-associated molecular patterns that activate innate immune cascades to prime adaptive immune memory. Vectors based on adeno- and vaccinia-viruses (a poxvirus family member) come with highly efficient gene transduction characteristics and are amenable to large scale manufacturing^12,13^. Moreover, many years of vaccine research have underpinned their benign safety profiles and their robust immunogenicity potential. For TB vaccination strategies specifically, replication-deficient, *Mtb* antigen-expressing vaccine vectors based on chimpanzee-derived, respiratory adenovirus serotype 3 (ChAd3)^14^ and modified vaccinia virus Ankara (MVA) poxvirus^15,16^ have been evaluated. ChAd3 has the potential to elicit both cellular and humoral immune responses and, besides showing good safety, it will be encountered in human populations with relatively little pre-existing, inhibitory immunity^17–21^. Likewise, MVA has proven immunogenic and safe, in particular also in BCG vaccinees in TB endemic regions^22,23^.

While vaccine targets were initially selected by antigen screening for immunodominant response profiles in the TB exposed, more recent approaches are aiming at broader antigenic coverage by targeting a diverse set of antigens, of which the level of expression is associated with different stages of the pathogen in its interaction with the host. Typically, we discern active mycobacterial replication, dormancy, and resuscitation. Building on gene expression data of *Mtb* under different microenvironmental conditions in the post-genome era^24,25^, Aeras selected the cassette that included the following five antigen genes for incorporation into the ChAd3 and MVA vaccine vectors: antigen 85B (Ag85B), early secreted antigenic target-6 (ESAT6), Rv2626, Rv1733, and resuscitation promoting factor D (RpfD). Ag85B and ESAT6 are classical antigens that appear immunodominant in active TB and are associated with a replicative state of *Mtb*^26^. Rv2626 and Rv1733, on the other hand, are expressed under the control of the dormancy regulon and upregulated during a non-replicative state, whereas RpfD expression is most prominent upon resuscitation from this dormant state. The aforementioned five antigens were inserted as a fusion protein into replication-defective ChAd3 (ChAd3-5Ag) in a single reading frame after a single promoter, and into MVA (MVA-5Ag) with the expression of each vaccine antigen under control of an individual MVA promoter sequence.

Apart from the immune-modulating characteristics of the viral vehicles and the antigenic nature of the selected antigens, the route of administration will affect the outcome of immunization^27^. BCG, like most other vaccines, is typically applied parenterally. However, a growing body of preclinical evidence indicates that (local) mucosal application of BCG is beneficial and potentially maximizing the protective immune response against aerogenic *Mtb* infection^28–31^. Also, aerosolized administration of viral vector-based TB subunit vaccines on top of primary parenteral BCG vaccination showed improved (local) immunogenicity in rhesus macaques, albeit with variable efficacy^32–35^. In this context of local versus peripheral compartments of immunity and the pulmonary space as the relevant area of primary *Mtb* infection, heterologous parenteral–mucosal administration for prime-boost vaccination strategies appeals as a sensible push–pull strategy that may optimize the adaptive host response repertoire.

In the current study, we set out to investigate systemic parenteral and pulmonary mucosal administration strategies for heterologous prime-boost vaccination with ChAd3-5Ag and MVA-5Ag in non-human primates (NHP). We interrogated the immunogenicity as well as protective efficacy of the selected vaccine dose using highly susceptible Indian-type rhesus macaques as a stringent NHP test condition. One group of animals received intradermal BCG as a benchmark vaccine and another was left untreated as non-vaccinated controls. In four investigational immunization strategies, the ChAd3-5Ag vaccine candidate was administered twice for homologous prime-boost vaccination, whereas the MVA-5Ag candidate was either or not applied as heterologous booster. In one out of four groups, the complete ChAd-ChAd-MVA array was given parenterally (CC^IM^M^ID^), whereas another group received the same regimen through the mucosal route only (CC^AE^M^AE^). A heterologous administration approach combined parenteral ChAd3-5Ag prime-boosting with mucosal MVA-5Ag boosting (CC^IM^M^AE^), and a group receiving a homologous prime-boost with ChAd3-5Ag mucosally only (CC^AE^) was included to disentangle the effect of additional MVA-5Ag vaccination. Immune responses were recorded both systemically and locally in the lung lumen. Protective efficacy was assessed by standard readouts of pathological involvement, of mycobacterial persistence in specific organs, and by ^18^F-FDG-based positron emission tomography–computed tomography (PET–CT) imaging.

## Results

### Study design and vaccine dosing

**Fig. 1:**
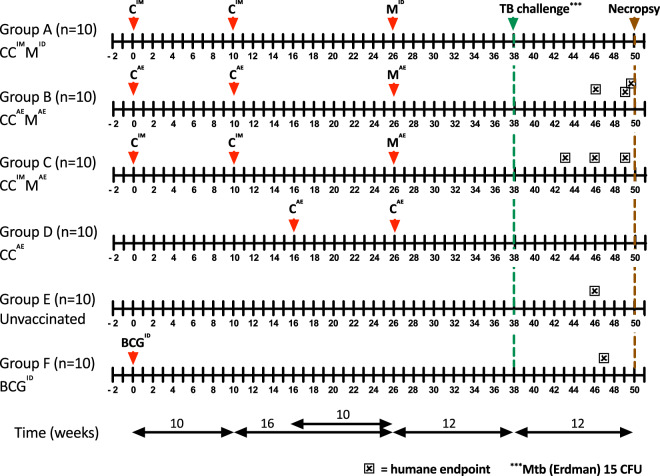
Study design. Schematic representation of the timeline of immunizations in the different experimental vaccination strategies, Mtb challenge, and necropsy. Animals reaching a humane endpoint are individually marked (☒) along the challenge phase.

Toward investigating heterologous ChAd3- and MVA-based prime-boost vaccination by parenteral and mucosal administration routes, we designed a study with four investigational and two control arms. The investigational strategies comprised the following groups: (A) a fully parenteral vaccination strategy of ChAd3-5Ag priming and boosting intramuscularly, and subsequent heterologous MVA-5Ag boosting intradermally (CC^IM^M^ID^), (B) the same regime but with respiratory aerosol administration of both vaccines (CC^AE^M^AE^), (C) the same regime again, but with parenteral ChAd3-5Ag prime-boosting intramuscularly and respiratory aerosol MVA-5Ag boosting (CC^IM^M^AE^), and (D) respiratory aerosol ChAd3-5Ag priming and boosting only (with no further MVA-5Ag administration) (CC^AE^). In all groups, the first, homologous ChAd3-5Ag booster was applied 10 weeks after primary vaccination; the second, heterologous MVA-5Ag booster 16 weeks after the ChAd3-5Ag booster. The vaccination scheme of group D (CC^AE^) was aligned such that in all investigational groups infectious challenge was delivered 12 weeks after the last vaccination event. Non-vaccinated animals were incorporated in group E as controls for immune analyses and protective efficacy after challenge. Group F, receiving a standard human dose of BCG intradermally at primary vaccination and infectious challenge 38 weeks later, served as a BCG benchmark control. The study design is depicted schematically in Fig. 1.

A total of highly outbred 60 healthy male rhesus monkeys (see Supplementary Table 1) that by interferon-gamma (IFN-γ) ELISPOT assay appeared free of pre-existing anti-mycobacterial immunity, were selected and stratified over 6 groups of *N* = 10 animals, thereby ensuring similar age and weight distribution over the groups (Supplementary Table 1). Treatment (A–F) was assigned to the groups randomly. Because of logistical limitations associated with animal handling, sampling, and downstream assays, animals were stratified over four stacks (two stacks of 14 and two stacks of 16 animals) with a similar representation of each treatment group in every stack (Supplementary Table 1). Stack 1–4 were undergoing handling, sampling, and assaying according to the study plan at 1-week intervals, with fresh preparation from stock of vaccine and infectious challenge inoculum every week.

**Fig. 2:**
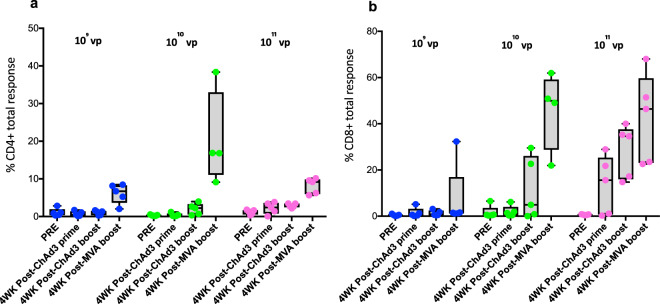
Antigen-specific CD4+ and CD8+ T cell in the lung measured for different ChAd 3-5Ag vaccine doses. **a** Percentage of CD4+ or **b** CD8+ T cells from lung washes (BAL), producing either IFN-γ, TNF-α, and/or IL-2 (in total) after antigen-specific *ex vivo* stimulation with peptide pools of individual antigens contained in the respective viral vector constructs. BAL samples were collected before vaccination (PRE), 4 weeks after ChAd3-5Ag priming (study week WK4), ChAd3-5Ag boosting (study week WK14) and MVATG18633 (10^8^ PFU) boosting (study week Wk28). vp viral particles. The box extends from the 25th to 75th percentiles. The line in the middle signifies the median and the whiskers include the smallest value up to the largest.

A dose-ranging pilot study in rhesus macaques was performed in order to select the ChAd3-5Ag dose. ChAd3-5Ag was delivered twice, using the Investigational eFlow® Technology nebulizer system (PARI Pharma GmbH, Munich, Germany), administering 10^9^, 10^10^, or 10^11^ vp, followed by heterologous aerosol boosting with 10^8^ PFU of MVATG18633, using the same time schedule as described above for the present infection experiment. Vaccine antigen-specific immunity was monitored by flow cytometric analysis of bronchoalveolar lavage cells (BAL; Fig. 2) and blood (PBMC; Supplementary Fig. 1) before and 4 weeks after each of the three vaccination events. Based on the analysis of the local immune response we observed that production of IFN-γ, tumor necrosis factor-alpha (TNF-α), and/or interleukin-2 (IL-2) in CD4+ and CD8+ T lymphocytes was optimal after prime-boosting with 10^10^ vp of ChAd3-5Ag (this equals 8.1 × 10^7^ FFU) (Fig. 2).

### Immunogenicity of experimental vaccines

**Fig. 3:**
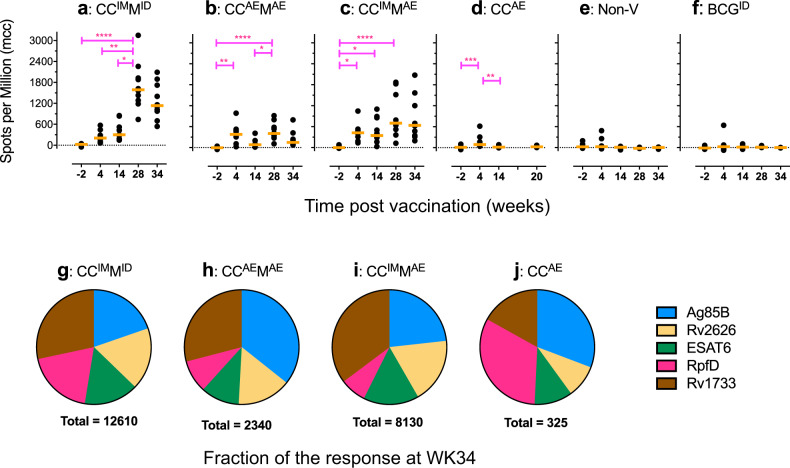
Peripheral Ag-specific IFN-γ response analysis along the immunization phase. Longitudinal Ag-specific IFN-γ production is determined by specific ELISpot, using freshly isolated PBMC and the vaccine-specific antigens contained in the vaccine vectors for in vitro recall stimulation. **a**–**f** IFN-γ response levels are shown before the start of the immunization (WK-2), 4 weeks after ChAd3-5Ag priming (WK04) and ChAd3-boosting (WK14), and 2 weeks after boosting with MVA-5Ag (WK28). WK34 indicates the last time point before challenging with *Mtb* Erdman in study week 38 (which corresponds to 20 weeks after ChAd3 priming in group D). Response levels are expressed as Spots per Million cells after (culture) medium control correction (mcc). Horizontal (orange) lines indicate median group levels. Statistical significance between timepoints (by Kruskal–Wallis with Dunn’s correction for multiple analysis) is indicated as follows: *p < 0.05; **p < 0.01; ***p < 0.001; ****p < 0.0001. **g**–**j** The proportion of each of the five vaccine antigens contributing to the total IFN-γ response at WK34 (and WK18 for group D) is displayed for each of the four vaccine groups from left to right. The “Total” number indicated below the pie charts represent the sum of specific spots for the 5 TB Ag.

During the immunization phase of the current vaccination–infection experiment, vaccine take was monitored by specific ELISpot assay, measuring IFN-γ-producing cells after in vitro recall stimulation of PBMC with peptide pools of the five respective vaccine antigens. Four weeks after primary vaccination with ChAd3-5Ag, either by intramuscular or by aerosol delivery, the summed IFN-γ response to all five vaccine antigens together was elevated in all vaccine groups A–D, but not in the non-vaccinated and BCG control groups (Fig. 3). This peripheral primary IFN-γ response was not further enhanced by intramuscular ChAd3-5Ag booster vaccination; 4 weeks after boosting we found a similar frequency of IFN-γ secreting cells. However, 4 weeks after aerosol ChAd3-5Ag boosting, both in groups B and D, the IFN-γ response was found decreased relative to the level after primary vaccination. When measuring neutralizing antibodies (NAbs) against the ChAd3 vector in serum, low titers of neutralizing antibodies were detected before primary immunization (Supplementary Fig. 2), which is in line with studies in humans^19,20,36^. These NAbs were most prominently increased after intramuscular ChAd3 (Group A and C; C^IM^), with an average relative neutralization of 59.2% (±20.2) after intramuscular priming versus 18.1% (±18.4) after aerosol priming (Supplementary Fig. 3). Neutralization after homologous intramuscular boosting at week 10 was 99.3% (±0.72) versus 27.7% (±25.2) after aerosol boosting (Supplementary Fig. 2). The increase of peripheral NAb against the vector after primary vaccination may (in part) explain the lack of a boosting effect.

Heterologous, intradermal MVA-5Ag boosting significantly enhanced the peripheral IFN-γ response induced by CC^IM^ (group A; Fig. 3a). When given through aerosol after CC^IM^, this booster effect of MVA-5Ag was less prominent and not statistically significant (group C; Fig. 3c). Although aerosol MVA-5Ag boosting after aerosol CC^AE^ prime-boosting did lead to statistically significant enhancement of the peripheral IFN-γ response, the absolute response level did not exceed that of the C^AE^ primary vaccination (WK4; group B; Fig. 3b). Six weeks later (that is 8 weeks after heterologous boosting and 4 weeks prior to infectious challenge) the vaccine antigen-specific IFN-γ response is somewhat waning in groups A, B, and C, yet still significantly elevated over the response prior to vaccination (WK-2). The highest peripheral responses are ultimately found upon full parenteral vaccination, CC^IM^M^ID^ (group A), while the lowest peripheral responses are registered with the mucosal vaccination regimes CC^AE^M^AE^ and CC^AE^, groups B and D, respectively (Fig. 3b, d).

The pie charts presented in Fig. 3g–j depict the relative contribution of the individual vaccine antigens to the summed, peripheral IFN-γ response. They demonstrate that all antigens contributed to the overall IFN-γ response without any clear immunodominance but with some variation between the vaccination regimes.

As another parameter of vaccine take and immunogenicity we measured cell proliferation in PBMC after in vitro recall stimulation with either of the five vaccine antigens. In line with the findings from the IFN-γ ELISpot assay, the sum of proliferation indexes obtained with each of the five antigens increased with every vaccination event (Supplementary Fig. 3a–d), yet remained at baseline throughout in non-vaccinated and BCG control groups (Supplementary Fig. 3e, f). Ultimately, 8 weeks after final vaccination and 4 weeks before infectious challenge, highest proliferation is observed after parenteral prime-boost immunization in groups A and C, CC^IM^M^ID^ and CC^IM^M^AE^, respectively.

Breaking down the relative contribution of the individual vaccine antigens to this proliferative response shows that all contribute without clear immunodominance in any of the investigational vaccine groups (Supplementary Fig. 3g–j).

In line with the vaccine antigen-specific responses, PPD-specific IFN-γ release in PBMC was most prominent in groups A (CC^IM^M^ID^) and C (CC^IM^M^AE^), somewhat lower in group B (CC^AE^M^AE^), but absolutely the lowest by group medians in D (CC^AE^) (Supplementary Fig. 4a–d). The highest PPD-specific response, as expected, was found upon BCG vaccination, while non-vaccinated controls remained negative throughout (Supplementary Fig. 4f, e, respectively). Measuring PPD-specific cell proliferation, yielded a comparable hierarchy of response levels amongst the investigational vaccine regimes as well as for the vaccine antigens, albeit that responses were only weakly increased over background (Supplementary Fig. 4g–j). At the same time and expectedly, PPD-specific cell proliferation was most significantly and robustly elevated in the BCG control group F (Supplementary Fig. 4l).

### Antigen-specific production of classical Th1 cytokines by CD4 and CD8 T cells

**Fig. 4:**
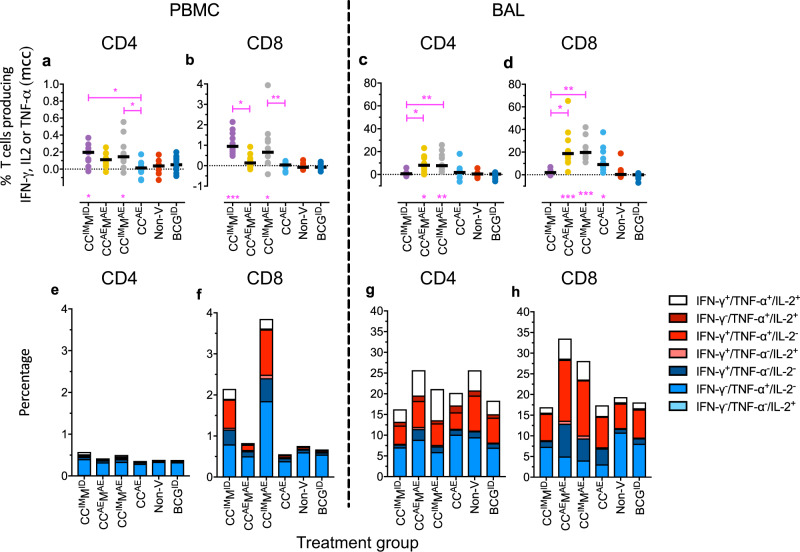
Peripheral and local immune response analysis by flow cytometry. Cross-sectional analysis of classical Th1 cytokine production (IFN-γ, TNF-α, or IL-2) per treatment group at week 34, before infectious challenge: **a**, **c** by CD4+ or **b**, **d** CD8+ T cells, **a**, **b** in PBMC and **c**, **d** in BAL samples, respectively. Freshly isolated cells were stimulated in vitro with specific antigens as they are expressed by the respective vaccine vectors, and frequencies of cytokine positivity are summed for all five antigens. Horizontal (black) lines indicate group medians. **e**, **f** Stacked bar graphs showing the WK34 frequencies of multifunctional cytokine-positive subsets per treatment group (single, double, and triple positive subsets for IFN-γ, TNF-α, or IL-2, according to the color legend), **e**, **g** for CD4+ and **f**, **h** CD8+ T cells analyzed in **e**, **f** PBMC and **g**, **h** BAL samples. Statistical significance (by Kruskal–Wallis with Dunn’s correction for multiple analysis) of total cytokine response levels in comparison to the non-vaccinated (Non-V) control group is indicated per vaccine group above the *x*-axis (in magenta symbols). Significance between groups is indicated where it applies by connecting lines (in magenta) *p*-Values are summarized as follows: *p < 0.05; **p < 0.01; ***p < 0.001; ****p < 0.0001.

To further characterize vaccine antigen-specific T lymphocyte function, the production of IFN-γ, TNF-α, and IL-2 was analyzed by flow cytometry systemically (PBMC), as well as locally (bronchoalveolar lavage (BAL) cells). To this end, we assessed cytokine production by CD4+ and CD8+ T lymphocytes after in vitro stimulation with peptide pools of any of the five vaccine antigens 8 weeks after final vaccination (4 weeks before infectious challenge). In the periphery, the (summed) frequencies of CD4+ or CD8+ T lymphocytes producing any of the three cytokines were increased significantly as compared to the non-vaccinated group, only with the vaccination regimes comprising parenteral, intramuscular CC^IM^ prime-boosting (groups A and C; Fig. 4a, b for CD4+ and CD8+, respectively).

These responses in PBMC from group B, CC^AE^M^AE^, were also elevated, but without statistical significance. Group D, CC^AE^, was indistinguishable from non-vaccinated controls (or the BCG control group for that matter), in the peripheral compartment. The same response pattern appears when plotting stacked bars representing the functional subsets of single, double, and triple cytokine-positive CD4+ and CD8+ T cells (Fig. 4e, f). Triple cytokine-positive CD8+ cells producing IFN-γ, TNF-α, and IL-2 simultaneously are only observed after CC^IM^M^ID^ and CC^IM^M^AE^ vaccination (groups A and C, Fig. 4e, f). Some distinctive double-positive CD4+ and CD8+ cell responses were found after CC^IM^M^AE^, group C (Fig. 4f).

In BAL cytokine-producing CD4+ T lymphocytes were only observed after mucosal MVA-5Ag (groups B and C, Fig. 4c). Cytokine-producing CD8+ T cells in BAL were significantly induced in either of the regimes with aerosol vaccine administration: CC^IM^M^AE^, CC^AE^M^AE^ as well as CC^AE^ (groups B, C, and D, Fig. 4d). Group A, with full parenteral immunization (CC^IM^M^ID^), was indistinguishable by BAL cell analysis from non-vaccinated controls (or the BCG control group for that matter). Assessing single, double, and triple cytokine-producing subsets separately (Fig. 4g, h) did not reveal a clear functional shift by the investigational vaccination regimes, although the highest frequency of triple positive, IFN-γ+, TNF-α+, and IL-2+-producing CD8+ T cells in particular, seemed to appear upon mucosal MVA-5Ag vaccination (groups B and C, Fig. 4h). (Lymphocytes in the pulmonary lumen per se appear with a multi-functional double and triple cytokine producing phenotype).

Altogether, functional T lymphocyte assays demonstrate successful vaccine take in all investigational (as well as BCG control) immunization groups. By and large, quantity and quality of systemic versus local pulmonary immune responses differentially match with parenteral (IM/ID) versus respiratory mucosal (AE) vaccination strategies, respectively. While homologous, mucosal prime-boosting with CC^AE^ only (group D) appears the least immunogenic, MVA-5Ag boosting by either administration route significantly adds to the vaccine-specific immune response.

### Protective efficacy of the investigational vaccine strategies

To assess the protective efficacy of the investigational vaccination strategies all animals including controls were challenged by endobronchial instillation of a target dose of 15 CFU of *Mtb* strain Erdman 12 weeks after final immunization (study week 38). Quality control analysis of inoculum preparations per stack and retrospect analysis per stack of eventual gross pathological involvement (see below), corroborates that similar challenge doses were applied over time to all stacks (Supplementary Fig. 5a, b). Antigen-specific IFN-γ signals in PBMC from non-vaccinated controls after challenge were most prominent upon in vitro recall stimulation with ESAT6 or Ag85B (Supplementary Fig. 6), and illustrate de novo induction of *Mtb*-specific immune responses. The investigational groups showed significant enhancement of the peripheral IFN-γ response post-challenge against ESAT6 only. BAL cells were not collected during the infection-phase and, thus, local pulmonary immune response characteristics post-infection were not available.

**Fig. 5:**
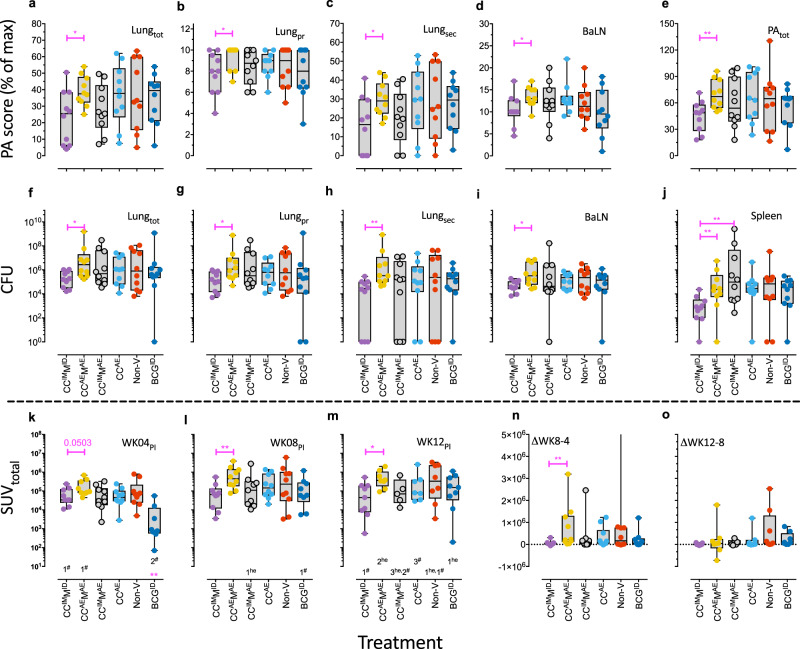
Readouts of vaccine efficacy. **a**–**e** Pathology scores (PA) at necropsy per treatment group in arbitrary units. **a** Total lung score (Lung_tot_); **b** primary lung lobe score (Lung_pr_: the lobe where the inoculum was instilled); **c** secondary lung lobe score (Lung_sec_: remaining 6 lung lobes, added up as a measure of pulmonary dissemination); **d** lung draining (bronchoalveolar) LN score (BaLN), and **e** total PA scores (PA_tot_: all PA scores added together). **f**–**j** The mycobacterial burden was enumerated by serial, quantitative plating of tissue sample homogenates and calculated for **f** Lung_tot_; **g** Lung_pr_; **h** Lung_sec_; **i** BaLN, and **j** spleen. **k**–**o** Total lung [^18^F]-FDG uptake was measured by PET–CT along the infection phase and is expressed here as SUV_total_ (total standardized uptake value): **k** at WK04 (significance compared to Non-v control is indicated by magenta double asterisk above the *x*-axis); **l** WK08; **m** WK12 relative to infectious challenge. Disease progression is shown by plotting the difference in SUV values **n** from WK4 to 8 (∆WK8-4) and **o** from WK8 to 12 (∆WK12-8). The number just above the *x*-axis indicates the number of images not obtained at that point in time due to human endpoint (he) or technical failure (#). Mann–Whitney tests were performed to compare complete parenteral immunization versus complete mucosal immunization (CC^IM^M^IM^ versus CC^AE^M^AE^). Significance is indicated by connecting lines in magenta (*p < 0.05; **p < 0.01).

At endpoint, either 12 weeks post-infection by study plan or any earlier when humane endpoint criteria applied, animals were euthanized for post-mortem evaluation of TB disease and protective vaccine effects. Humane endpoints occurred in 8 out of 60 animals from week 5 after challenge onward and affected 3 out of 10 from groups B and C and 1 out of 10 from groups E and F (see Fig. 1). By scoring macroscopic pathological involvement none of the investigational treatment groups showed a significant signal of protection relative to non-vaccinated controls, neither by summed total lung pathology scores (Fig. 5a; Lung_tot_) nor by scoring the primary lung lobe targeted by bronchoscopic instillation (the left caudal lobe) or the other lobes together (as a measure of pulmonary dissemination) (Fig. 5b, Lung_pr_, and Fig. 5c, Lung_sec_, respectively). The variation of lung pathology in the non-vaccinated controls of this outbred cohort was (expectedly) large, and also standard intradermal BCG failed to reduce TB pathology, which is not unprecedented in this model and confirming its stringency^30^. Also, by bronchoalveolar lymph node scores (Fig. 5d; BaLN) or by total pathology scores as the sum of lung, bronchoalveolar lymph node and extra-thoracic organ (typically spleen, liver, and kidney) involvement together (Fig. 5e; PA_tot_), no impact of prior vaccination could be demonstrated. However, significantly lower pathology scores by all these aspects of primary and disseminated disease, were evident in group A, CC^IM^M^ID^, with parenteral vaccinations only, when compared to group B, CC^AE^M^AE^, which received respiratory mucosal immunization throughout (Fig. 5a–e).

In addition, *Mtb* bacterial load was determined in the various tissues by plating serial dilutions of tissue homogenates. Overall and in line with pathology scores, neither by total (summed) lung CFU, nor by mycobacterial enumeration from primary targeted or secondary lung lobes separately, nor from bronchoalveolar lymph nodes or spleen (Fig. 5f–j), any protective effect of the vaccine regimes relative to non-vaccinated controls became apparent. Again, and in line with pathology scores, bacterial burden in group A, CC^IM^M^ID^ (parenteral vaccination only), was significantly lower compared to group B, CC^AE^M^AE^ (respiratory mucosal immunization only) (Fig. 5f–j).

The disease appears more contained to the site of infection in group A than in group B, since a few animals in group A (4/10) showed no dissemination to the secondary lung lobes (Fig. 5h), while all the animals in group B had detectable CFU.

However, it has to be noted that the Non-V control group also showed no detectable bacteria in the secondary lung lobes of 3/10 animals. It is interesting to observe that the parenteral strategy showed the same trend of reduced bacterial burden in BaLN (Fig. 5i) and spleen (Fig. 5j) compared to the mucosal immunization strategy.

Beside assessing pathological and bacteriological parameters at endpoint, we also monitored TB disease development over time by pulmonary PET–CT scanning at 4, 8, and 12 weeks post-infection. To this end, we used ^18^F-FDG as a tracer of TB lesion-associated metabolic activity (read: inflammation). Yet, at no point after infection standard uptake values of ^18^F-FDG revealed any protective effect by the investigational vaccines as these values were not significantly different from those of the non-vaccinated control group (Fig. 5k–m). Prior standard BCG vaccination, however, resulted in a significantly reduced PET signal by week 4 after infection (Fig. 5k), which can be interpreted as a delayed onset of progressive inflammatory disease as it was nullified by week 8 (Fig. 5l). Again, and in line with pathology and bacteriology results, at all time points PET–CT signals were significantly lower in group A, CC^IM^M^ID^, compared to group B, CC^AE^M^AE^ (Fig. 5k–m).

Plotting the changes in standard uptake values of ^18^F-FDG from week 4 to week 8 and from week 8 to week 12 (Fig. 5n, o, respectively), show progressive disease until week 8 in all but group A, CC^IM^M^ID^ (complete parenteral vaccination). Based on the available data the PET signal remained stable after week 8 for the other investigational vaccine groups (Fig. 5o; ∆WK12-8). Most prominent disease progression from week 4 to 8 is measured in group B, CC^AE^M^AE^ (complete mucosal vaccination), which is statistically higher than group A.

By calculating non-parametric Spearman’s *ρ* we confirmed strong and highly significant correlations between pathology scores and mycobacterial burden of the lung, and between either of these two parameters and ^18^F-FDG PET signals over time (Supplementary Fig. 7). We did not identify any correlates when analyzing different immune response parameters (proliferation, IFN-γ production, and T cell responses) at week 34 versus disease readouts (pathology, bacterial load, and PET signals; Supplementary Fig. 8). In addition, we did not identify MHC Class I haplotypes (given in Supplementary Table 2 for each animal) that could be associated with increased/decreased bacterial load, as a surrogate marker for protection (Supplementary Fig. 9).

## Discussion

In this study, we evaluated four prime-boost vaccination strategies, exploring different routes of administration with two viral vectored vaccines encoding five TB antigen. Four investigational vaccination strategies combining the viral vectors ChAd3-5Ag (homologous prime-boost) and MVA-5Ag (heterologous boost) given parenterally by IM or ID (CC^IM^M^ID^), mucosally by aerosol (CC^AE^M^AE^), through a heterologous administration (CC^IM^M^AE^) or mucosal ChAd3-5Ag (CC^AE^) only, were tested as stand-alone strategies in non-BCG^ID^-primed animals. Despite the detection of local and peripheral antigen-specific immune responses, varying with route of administration, none of the four investigational vaccination strategies showed protection compared to the non-vaccinated control group. Interestingly, we observed less disease after a complete parenteral vaccination strategy (CC^IM^M^ID^) compared to a complete mucosal vaccination strategy (CC^AE^M^AE^).

The rhesus macaque model using the virulent *Mtb* Erdman for infection is a very stringent model of TB for testing new vaccine strategies. In this model, the effect of standard intradermal BCG^ID^ vaccination has been variable and, in this study, not significantly different than the nonvaccinated control (Fig. 5). However, we^28,30^ and others^37^ have demonstrated that signals of protection of TB disease and protection of infection can be obtained, justifying the use of this model for evaluation of vaccine efficacy.

Although antigen-specific immune responses were detected, we can only speculate on possible explanations for the lack of efficacy with the vaccination strategies tested in the current study. The following issues might have impacted on the outcome: vaccine dose by aerosol administration, the breadth of the immune response (number of antigens included in the vaccines), class of the immune response (anti-viral type-I IFN), non-specific effects of viral vectors on innate immune training, and persistence of the vaccine vectors. These issues will be discussed below.

First, when vaccinating parenterally by IM or ID injection, dosing is robust. However, with the hand-held nebulizer that was used in this study for aerosol vaccination the vaccine dose is less well controlled. In particular, the nebulizer mask is equipped with open vents to prevent pressure build-up during vaccine administration, resulting in substantial loss of nebulized vaccine into the immediate environment. Furthermore, differences in individual respiratory depth and rhythm affect the number of viral particles inhaled. So, the group that underwent full aerosol vaccination (group B) likely received a lower dose compared to the group that received parenteral vaccination only (group A). Although we aimed to elicit more effective pulmonary immunity, the aerosol administration strategy negatively affected the dosing and, thus, possibly vaccine efficacy.

Secondly, the number of antigens included in the vaccines determines the breadth of the immune response against TB. The five TB antigens that were included in the MVA-5Ag and ChAd3-5Ag vector in the current study and that cover different phases of the *Mtb* lifecycle, may have been limited in their capacity to mount an effective immune response. The fact that, based on our assays/analyses, we could detect immune responses specific for all encoded antigens indicates that these constructs are immunogenic. Notwithstanding the impact of the vector itself on the ensuing immune response (see below), a CMV vectored vaccine containing a fusion of three similar antigens (ESAT6, Rv2626, and RpfD) but also three additional antigens not included in our constructs (Ag85A, Rv3407, and RpfA), did confer significant protection in rhesus monkeys^37^. In the context of therapeutic vaccination in mice, a MVA vectored vaccine containing 10 TB antigens reduced the bacterial burden in combination with antibiotic treatment and prevented relapse of the disease^15^. Recently, adjuvated M72, containing only two antigens, Mtb32A and Mtb39A (encoded by Rv0125 and Rv1196) has been shown to prevent disease in a Phase 2b clinical trial^38,39^. Although broadening the antigen-specificity of a vaccine-induced response may seem beneficial, data do suggest that only 2–6 antigens in a subunit vaccine may be sufficient to control TB. It would remain interesting to further analyze in detail against which antigenic determinants the immune responses were directed, but this was beyond the scope of this study. Hence, among reasons possibly associated with vaccine failure to protect, dominance of some vaccine antigens/epitopes over others may have played a role in directing the most robust responses towards antigens/epitopes less important for protection.

Thirdly, whether mucosal administration outperforms standard parenteral administration strategies may depend on the nature of the vaccine used. Despite efficient gene transfer characteristics, potent immunogenicity and good safety profiles, using viral vectors as carriers for mycobacterial antigens might result in parallel induction of anti-viral immunity, which may be suboptimal or even detrimental for an effective response against TB. It is known that the type I IFN-dependent pathway that can be triggered by viral infection, impairs the control of *Mtb*^40–42^.

In addition, the so-called non-specific effects of vaccines by modulating innate immune responses may impact on the host response beyond the nominal induction of adaptive antigen-specific lymphocyte responses. In a recent paper by Blok et al.^43^, it was demonstrated in vitro that monocytes incubated with the recombinant MVA vector (and not the parental Vaccinia strain) induced innate immune tolerance, resulting in an inhibition of pro-inflammatory cytokine production after a second heterologous stimulation. Thus, one may speculate that local administration of MVA-5Ag as a final booster, compromises protective immunity. Whether the recombinant ChAd3-5Ag might have such an inhibiting effect on the local innate immune environment is unknown. In a recent paper by Yao et al.^44^, no difference was observed between a recombinant adenovector and the wildtype parental adenovirus in the induction of long-lasting trained immunity mediating anti-bacterial protection in murine alveolar macrophages. Yet, data by Darrah et al.^35^ suggest that combined AE/IM boosting with an empty Adeno 5 vector on top of BCG priming performed worse than BCG alone.

Lastly, persisting stimulation of the immune response by the continued presence of the vaccine, has been suggested as a prerequisite for efficacious TB vaccination^45^. Hansen et al. proposed the persistence of the CMV-vectored TB vaccine as an explanatory characteristic for the protection they observed^37^. Of note, that study contained no empty CMV vector control, while CMV infection is known to drive epigenetic changes^46^ which might also contribute to protection against TB. The viral vectors used in the current study are replication-defective and likely cleared after neutralizing antibodies arise from immunization.

Taken together, the immune responses induced by the experimental vaccination strategies tested here lacked quantity and/or quality to prevent TB infection or disease in rhesus monkeys. The impact of viral vectors, used as carrier for the TB antigens, in combination with the route of vaccination on the local immune environment is complex and warrants further investigation.

## Methods

### Animals

#### Dose-ranging study

The dose-ranging pilot study was carried out in 15 Chinese-origin rhesus macaques, 5 animals per group (*Macaca mulatta*; 3.9–12.2 kg; Supplementary Table 3). RM care and experimental BIOQUAL protocol number 15-043 were approved by the Institutional Animal Care and Use Committee (IACUC) prior to the start of the study. The study was approved by the MaGil institutional review board (IRB). All ethical regulations regarding animal research were complied with. Animals were housed and cared for in accordance with local, state, federal, and institute policies in facilities accredited by the American Association for Accreditation of Laboratory Animal Care, under standards established in the Animal Welfare Act and the Guide for the Care and Use of Laboratory Animals. Animals were pair-housed at Bioqual, Inc. throughout the study and were monitored for physical health, food consumption, body weight, temperature, complete blood counts, and serum chemistries.

#### Efficacy study

The study was carried out in 60 healthy, Indian-type, purpose-bred, pedigreed, male rhesus monkeys from the BPRC colony (RMs, *M. mulatta*; 5.99–13.50 kg; Supplementary Table 1). MHC Class I and II genotype of animals in the efficacy study are provided in Supplementary Table 2.

All RMs were screened to be negative for pre-existing immunity against mycobacterial antigens as determined by interferon-gamma (IFN-γ) ELISpot assay after in vitro recall stimulation with Purified Protein Derivate (PPD) of *M. tuberculosis*, *Mycobacterium* *avium*, and *M. bovis*. The RMs were housed in socially compatible pairs at the Biomedical Primate Research Centre (BPRC) in full compliance with the European Directive 2010/63/EU (animal biosafety level ABSL-3). The animals were offered a daily diet consisting of monkey food pellets (Hope Farms, Woerden, The Netherlands), fruit and vegetables of the season, and bread. Drinking water was available ad libitum via automatic water systems.

RM care and all experimental protocols and procedures were approved by the BPRC Institutional Animal Care and Use Committee (IACUC) prior to the start of the study under the Framework Application 726 and in full compliance with national and European legislation and guidelines. The study was approved (726subC and 726subD; 04-10-2016) by the Animal Welfare Body (AWB) of BPRC. The BPRC is accredited by the American Association for Accreditation of Laboratory Animal Care (AAALAC) and has an approved Assurance (A5509-1) for the care and use of animals on file at the National Institutes of Health (NIH).

### Animal procedures

The RMs were sedated with ketamine-HCl (10 mg/kg) for intradermal- and intramuscular vaccination and blood collection through venipuncture. For aerosol vaccine administration, bronchoalveolar lavage (BAL) and intrabronchial *Mtb* inoculation animals were sedated with ketamine-HCl (5 mg/kg) and medetomidine (0.04 mg/kg). RMs were sedated with ketamine-HCl (5 mg/kg) and medetomidine (0.04 mg/kg) after which positron emission tomography–computed tomography (PET–CT) procedures were performed under full isoflurane anesthesia. The *Mtb* inoculum was delivered to a segmental bronchus in the left caudal lung lobe using a bronchoscope. The RMs were challenged with 11–19 colony forming units (CFU) per inoculum with a targeted average dose of 15 CFU per inoculum in a volume of 3 ml of physiological saline solution. Due to the size of the cohort and logistical limitations, this study was executed in a stacked manner with animals divided into four stacks that were treated similarly at 1-week intervals. By stratification, each stack contained animals from each treatment group (see Supplementary Table 1). Cells from the pulmonary mucosa were recovered at specific time points by bronchoalveolar lavage (BAL), targeting the lower left lung lobe. Three volumes of 20 ml of prewarmed 0.9% saline solution were consecutively instilled and recovered. BAL fluid was harvested by centrifugation of BAL samples for 10 min at 400*g* after 100 μm filtration. Supernatant was subsequently decanted and stored at −80 °C pending further analysis. The BAL cell pellet was resuspended in Roswell Park Memorial Institute medium supplemented with 10% fetal calf serum (FCS), glutaMAX, and penicillin/streptomycin (from now on referred to as R10) and used in downstream assays freshly.

### Vaccines and vaccination

The recombinant chimpanzee adenovirus type-3 vector-based TB candidate vaccine (ChAd3-5Ag) was constructed at GSK (Rixensart, Belgium) as described previously for ChAd3-SIV^20^ but using the pChAd3 ΔE1,E4/Ad5orf6 backbone. A selected stable clone was amplified in 293-F cells and the virus was purified from the supernatant by cesium chloride gradient centrifugation (a one-step gradient, followed by a continuous gradient), passed over a 2 μm filter and stored at −80 °C until use. The recombinant Modified Vaccinia Ankara virus expressing the 5 *Mtb* Ag (MVA-5Ag) was constructed at Transgene (Illkirch-Graffenstaden, France)^15^. ESAT6 was placed under the control of the pB2R promoter while Rv1733 was inserted downstream the pH5R promoter. Rv2626 sequence was placed under the control of the pSE/L promoter and Ag85B was inserted downstream the p7.5 promoter. Finally, RpfD was under control of pA35R promoter. The recombinant MVA, MVATG18633, used in the dose-finding study, was administered at a dose at 10^8^ plaque-forming units (PFU)/dose/animal, encoded three full-length Ags and one partial Ag in common with ChAd3-5Ag. MVATG18633 encodes for ESAT6 under control of pB2R promoter, Rv1813 downstream the pH5R promoter, Rv2626 placed under control of pSE/L promoter, Ag85B downstream the p7.5K promoter and the RpfB (30-284)-RpfD (54-154) fusion protein.

In the dose-ranging study, all animals were seronegative for ChAd3 titers. Fifteen animals were randomized into three vaccine groups (*n* = 5 per group) and immunized by aerosol with 10^9^, 10^10^, or 10^11^ vp of ChAd3-5Ag twice, at 0 and 10 weeks. Sixteen weeks later (week 26), all animals received 10^8^ PFU of MVATG18633 via the aerosol route. BAL (Fig. 2) and PBMC (Supplementary Fig. 1) were monitored by flow cytometry for T cells responding to overlapping peptides corresponding to the five ChAd3-vectored mycobacterial antigens prior to immunization, 4 weeks after the first and second ChAd3 administration, and 4 weeks after the MVA boost. Cytokine responses shown are background-subtracted summed responses to the five peptide pools (2 μg/ml final).

Frozen aliquots of viral stocks of ChAd3-5Ag and MVATG18633 or MVA-5Ag were thawed and formulated and diluted in physiological salt solution and kept on ice (4 °C) just prior to administration. For aerosol administration, the investigational eFlow® Technology nebulizer system (PARI Pharma GmbH, Munich, Germany) was used. The dose of ChAd vector used for parenteral vaccination was based on previous experiments using the ChAd3 vector^47^. Briefly, ChAd3 was dosed in NHPs at 10^9^, 10^10^, 10^11^ vp and based on immunogenicity and reactogenicity, 10^10^ vp was selected. To establish its performance and for prewetting, the device was first loaded and operated with 1 ml of saline. Subsequently, the animals were immunized by aerosol administration of a vaccine dose in 1 ml. For parenteral administration ChAd3-5Ag (8.1 × 10^7^ foci forming units (FFU)/animal = 10^10^ viral particles (vp)/animal) was given intramuscularly (IM) in 1 ml of saline and MVA-5Ag (10^8^ PFU/animal) was given intradermally (ID) in 0.5 ml. Animals immunized with Bacillus Calmette-Guérin (BCG) strain Sofia (InterVax Ltd.) received a single standard adult human dose of 1.5–6.0 × 10^5^ CFU in 0.1 ml intradermally.

Quality control analysis was performed at each round on the virally vectored vaccine preparations. After thawing a consistent recovery of 17–41% of the expected concentration of ChAd3-5Ag was observed. For the final vaccination with MVA a recovery between 88 and 148% of the expected MVA concentration was observed.

#### FFU assay for ChAd3-5Ag formulation

Serial dilutions of ChAd3-5Ag were added to AD-293 (AD-293; Stratagene) cells for 1 h at 37 °C. Virus suspensions were removed and the cells were cultured for 46–48 h at 37 °C. AD-293 monolayers were washed and fixed with ice-cold methanol for 10–20 min at −20 °C. Fixed cells were stained with mouse anti-Adenovirus (Abcam, ab7428) and then incubated with goat anti-mouse IgG-HRP (Thermo Fisher). Viral foci were visualized with 3,3′,5,5′-tetramethylbenzidine liquid substrate (TMB, Mabtech). FFU were counted microscopically and ChAd3-5Ag titer was determined.

#### PFU assay for MVA-5Ag formulation

Serial dilutions of MVA-5Ag were added to DF-1 cells (ATCC, CRL-12203) and incubated for 45 min at RT. Then, a DMEM–agarose suspension was added to DF-1 cell cultures and solidified for 30 min at room temperature. Cultures were incubated for 72 h at 37 °C. Subsequently, a DMEM–agarose containing neutral red as a dye (0.33%, Sigma), was added to stain DF-1 cell monolayers and incubated for an additional 4 h at 37 °C until MVA viral plaques were visible. Plaques were enumerated visually from the most appropriate dilution (showing 5–100 plaques/well) and MVA titers were calculated in PFU/ml.

### Immunological assays

#### Reagents

For the immune assays described below, the following reagents were used for antigenic stimulation. Peptide pools of each of the five antigens included in the constructs are described as follows: for Ag85B, 15 µg/peptide/vial, 69 peptides in total (JPT, MA, USA, Lot: 070813BAR-1); for Rv2626, 15 µg/peptide/vial, 33 peptides in total (Biosynthesis Inc., TX, USA, Lot: T6455-1); for ESAT-6, 15 µg/peptide/vial, 21 peptides in total (JPT, MA, USA, Lot: 200814BAR-2); for RpfD, 15 µg/peptide/vial, 36 peptides in total (JPT, MA, USA, Lot: 12125); for Rv1733, 15 µg/peptide/vial, 50 peptides in total (JPT, MA, USA, Lot: 10741). All peptide pools consisted of 15-mers with 11aa overlap and were synthesized to 80% purity. These antigens were added to a final concentration of 1 µg/ml. Purified Protein Derivative of Tuberculin (PPD; batch T50, SSI, Denmark) was added at a final concentration of 5 µg/ml. As positive control stimuli, a mix of Phorbol 12-Myristate 13-Acetate (PMA; 50 ng/ml, Sigma) and Ionomycin (1 µg/ml, Sigma) was applied for ELISpot assay, Concanavalin A (ConA, 5 µg/ml, Sigma) for T cell proliferation assay (TCPA), and Staphylococcus enterotoxin B (SEB, 1 µg/ml; Sigma) for FACS. R10 complete medium was used as a negative control of stimulation and consisted of Roswell Park Memorial Institute (RPMI) supplemented with 10% FCS, glutamax, and penicillin/streptomycin.

#### IFN-γ ELISpot assay on peripheral blood mononuclear cells (PBMC)

A NHP-specific IFN-γ ELISpot assay was used on PBMC, according to the manufacturer protocol (U-CyTech, Utrecht), to determine the frequency of antigen-specific IFN-γ producing cells both in the vaccination phase (38 weeks) and infection phase (12 weeks). In brief, 200.000 freshly isolated PBMC were incubated in triplicate for 24 h with specified antigens or control stimuli. Subsequently, supernatant was collected and stored (−80 °C), and cells were transferred to specific anti-IFN-γ coated filter plates (PVDF, Millipore) for an additional overnight (18 h) incubation. Cells were discarded and membrane-bound IFN-γ was detected using biotinylated anti-IFN-γ antibody, streptavidin-horseradish peroxidase conjugate, and tetramethylbenzidine (TMB) substrate (the latter from MAbTech, Stockholm). Spots were quantified using an automated reader (AELVIS, Hannover).

#### T cell proliferation assay (TCPA)

Antigen-specific proliferation was determined through tritiated (^3^H) thymidine incorporation. In brief, 100.000 freshly isolated PBMC were incubated with specified antigens in triplicate in 96-well round-bottom microtiter plates for 72 h at 37 °C. Supernatant was collected and stored, and ^3^H-thymidine (0.5 µCi/well/25 µl) was added to the wells. Cells were incubated overnight in a humidified incubator at 37 °C. Cells were harvested onto a filter plate. After drying of the filter plate, MicroScint-E scintillation fluid (PerkinElmer) was added and incorporation of radiolabel as a measure of proliferation was determined on a TopCount (PerkinElmer) scintillation counter.

#### Immune cell phenotyping using flow cytometry

Cell type-specific, antigen-specific immune responses were determined at indicated timepoints by flow cytometry using BAL cells and PBMC. Cells were stimulated overnight in the presence of Golgiplug transport inhibitor (BD; Biosciences).

The following mAb staining T cell panel was used for the dose-ranging study: Intracellular staining: CD3^APC-Cy7^ (SP34-2, BD; cat# 557757, dilution 1:320); CD69^ECD^ (TP1.55.3, Beckman Coulter; cat# 6607110, dilution 1:50); IFN-γ^APC^ (B27, BD; cat# 554702, dilution 1:320), TNF-α^BV650^ (Mab11, BD; cat# 563418, dilution 1:25), IL-2^PE^ (MQ1-17H12, BD; cat# 544566, dilution 1:320); Surface staining: CD4^PE-Cy5.5^ (S3.5, Invitrogen; cat# MHCD0418, dilution 1:80), CD8^BV570^ (RPA-T8, Biolegend; cat# 301038, dilution 1:160), CD28^Cy5-PE^ (CD28.2, BD; cat# 555730, dilution 1:10), CD45RA^PE-Cy7^, (L48, BD; cat# 337167, dilution 1:200).

##### Gating strategy dose-ranging study

Fresh PBMC samples unstimulated (medium control) and PPD stimulated. (1) Gating on singlets; (2) Gating on lymphocytes (FSC/SSC); (3) Gating on live cells (L/D stain) versus CD3^+ve^ cells; (4) Gating on CD3^+ve^ cells and subsequent gating on CD4 versus CD8; (5) Gating on CD28 versus CD45RA to select for CD4 and CD8 memory T cells; (6) Subsequent gating on CD69 versus cytokines (IL-2/IFN-γ/TNF-α). See Supplementary Fig. 10.

The following mAb staining T cell panel was used for the efficacy study: Intracellular staining: CD3 ^AF700^ (SP34-2; BD; cat# 557917; 1:160), TNF-α^PE-CY7^ (Mab11; BD; cat# 624052; 1:10), IL-2^BV510^ (MQ1-17H12; BioLegend; cat# 500338; 1:40), IFN-γ^APC^ (4S.B3; BD; cat# 554702; 1:160). Surface staining: CD4^PerCP-Cy5.5^ (L200; BD; cat# 5324924; 1:320), CD8α^APC-H7^ (SK1; BD; cat# 641400; 1:80), CD14^BV421^ (M5E2; BD; cat# 565283; 1:80), CD20^BV421^ (2H7; BioLegend; cat# 302330; 1:320), CD45^BV786^ (D058-1283; BD; cat# 563861; 1:160).

##### Gating strategy efficacy study

Fresh PBMC samples unstimulated (medium control) and PPD stimulated. (1) Gating on CD45^+ve^ cells (including all leukocytes/SSC); (2) Gating on singlets; (3) Gating out death cells (L/D stain) and CD20^+ve^/CD14^+ve^ cells (excluding B cells and monocytes in one channel) versus CD3^+ve^ cells (including all T cells); (4) Gating on CD3^+ve^ cells and subsequent gating on CD4 versus CD8. (5) Boolean gating of any cytokine expression of IL-2/IFN-γ/TNF-α. See Supplementary Fig. 11.

#### Anti-ChAd neutralization assay

The assay measured infection of cells based on GFP expression. ChAd3-GFP virus were pre-incubated with titrated amounts (10×, 50×, and 200×) of serum for 1 h and then used for infection of AD293 cells. As control, virus only was added to the AD293 cells. After 1 h of incubation virus suspensions were removed and the cells were cultured for 46–48 h at 37 °C. The readout was the number of GFP+ cells measured by Guava Technology. The cut-off for each individual plate was determined by taken the average of “cell only” values +3× the standard deviation. “Cell-only” is the fluorescence of the AD293 cell-line alone (autofluorescence). Percentage neutralization was determined by virus + serum over virus-only values. The data depicted (Supplementary Fig. 2) are at 10× diluted serum.

### Imaging

Pre- and post-challenge PET–CT images (320 µm slices for CT and 800 µm for PET with a field of view (FOV) of 15 cm axial against 20 cm transaxial) were obtained using a preclinical MultiScan LFER 150 PET–CT scanner (Mediso Medical Imaging Systems, Budapest, Hungary) on anesthetized RMs in a BSL-3 imaging suite.

^18^F-FDG was used to visualize metabolic/inflammatory activity in the lungs. All CTs were acquired under isoflurane anesthesia with a breath hold at a positive end-expiratory pressure (PEEP) of 4 cm. Both CT scans before and after CT contrast injection were acquired using a semicircular, single FOV scan method, with an exposure of 90 ms and 1:4 binning, using 75 kVp, and 980 µA CT tube strength. For optimal granuloma detection, CT acquisitions after intravenous non-ionic iodinated CT contrast injection (Omnipaque 300, 2 ml/kg, 1–2 ml/s, GE Healthcare, Hoevelaken, Netherlands) were acquired after a delay of 70 s. To detect granulomas that were metabolically active, a 20 min static PET scan was acquired. Static PET data were also acquired under isoflurane anesthesia after 45 min uptake time of an intravenous injection of 100 MBq ^18^F-FDG with 1:9 coincidence mode and a time-window of 5 ns. Post-CT reconstruction was performed using Butterworth filter, with a convolution scatter correction considering 16 cm diameter of the RM and having a slice thickness and voxel size of 320 µm. List mode PET emission data were iteratively reconstructed (OSEM3D, 8 iterations and 9 subsets) into a single frame PET image voxel size of 800 µm. PET data were corrected for radioactive decay, scatter and random coincidences. In addition, attenuation correction was performed via the formation of a material map for which the CT without contrast was used.

PET–CT images of all RMs were acquired pre-challenge and on WK4, WK8, and WK12 (the latter, 2 days before necropsy). Scans were analyzed by the Central Reading Facility at the University of Pittsburgh. Analysts were blinded to treatment of the subject other than the infectious challenge dose and time of PET–CT recording relative to infection. All images were analyzed using the OsiriX® DICOM Viewer (Pixmeo, Bernex, Switzerland). For analysis, three CT slices and two PET slices were combined. The number of granulomas were counted on CT while total lung ^18^F-FDG uptake and lymph node activity by PET was quantified according to previously established methods^48^. Metabolic activity was expressed as total standard uptake value (SUV_total_).

### Necropsy and pathology scoring

The humane endpoint criteria for removing RMs with overt TB from the study, were as follows: (i) marked lethargy, (ii) severe dyspnea at rest or after exercise, (iii) sustained weight loss (>10% in 3 weeks; >20% over any time course), (iv) sustained poor appetite, indicated late-stage TB, and animals were immediately euthanized and taken to necropsy. RMs that remained clinically well were scheduled by protocol for euthanasia and necropsy at week 12 after infectious challenge. Eight animals reached a humane endpoint before week 12. At humane endpoint or at week 12, a limited volume of blood (<45 ml) was collected from the RMs before euthanasia with sodium pentobarbital (>50 mg/kg).

The necropsy procedure included complete gross pathological evaluation of abdominal organs and tissues. Macroscopic granulomas in the liver, spleen, and kidney were counted, measured, and photographed and given a numeric point value score using a semi-quantitative grading system (Supplementary Tables 4.1 and 4.2). Axillar, inguinal LN, and the spleen were collected for detailed analysis. The spleen was used for mononuclear cell isolation and *Mtb* quantification. The pleura and thoracic wall were examined after entering the thoracic cavity. The thoracic viscera were removed en bloc and then transferred to a sterile cutting board for further examination, dissection, and scoring. Individual lung lobes were dissected, weighed and photographed. The bronchoalveolar lymph nodes LNs (BaLNs) were weighed and photographed. Samples of the BaLN were collected for quantitative *Mtb* culture. Individual lung lobes were cut in serial slices and macroscopic granulomas were counted, measured, photographed, and scored. The lobe targeted for infection was designated the primary lung lobe (typically the left caudal lung lobe). The remaining 6 lobes were collectively designated secondary lobes. Samples for *Mtb* culture were collected from the primary and secondary lung lobe, the BaLN and the spleen. After initial homogenization, random samples of primary and secondary lung lobes (of 2–3 g) were collected in 4 ml RPMI in Gentlemax C-tubes for more strenuous homogenization. A random sample of spleen and BaLN, both after securing representative samples for histological evaluation, were collected in RPMI (10 and 8 ml, respectively) and strained through a sieve. Homogenates of spleen and BaLN were further processed in Gentlemax C-tubes. The weight of organs and samples was recorded in order to calculate dilution factor and bacterial burden afterwards. All samples were stored at −80 °C prior to analysis at later timepoint.

### Bacteriology

#### *Mtb* inoculation dose formulation

Three vials of *Mtb* strain Erdman K01 (BEI Resources; lot K1/LI409, manufacturing date April 2009) were thawed and pooled per challenge event (to minimize possible “per vial” variation), serially diluted and plated on standard medium for enumeration and confirmation of the challenge dose. Plating was performed in 10 replicates by manual plating (100 µl/plate). CFU were counted (21–28 days after plating) using a Scan® 4000 colony counter.

#### Determination of bacterial load in tissue

Homogenate samples (±2 ml) of primary and secondary lung lobe tissue were thawed. Debris was removed by pulse centrifugation. Primary and secondary lung, and BaLN samples were serially diluted and plated using the Easy Spiral Dilute™ (Interscience, France), all in duplicate. From the spleen undiluted homogenate samples of 100 µl were plated in duplicate. All plates were incubated at 37 °C, and *Mtb* colonies were enumerated 21 and 35 days later with a Scan 4000 plate reader.

### Statistical analysis

Statistical analyses were conducted using GraphPad Prism 7 software (version 7.0d; GraphPad Software, La Jolla, CA). For group comparison analysis, a non-parametric rank test (Kruskal–Wallis) was performed with post-hoc Dunn’s correction for multiple comparison, to compare the vaccine treatment groups to the non-vaccinated group. Subsequently, a Kruskal–Wallis test with post-hoc Dunn’s correction for multiple comparison was performed among the four experimental groups to establish whether one strategy was statistically better than others. A Mann–Whitney test was performed to compare TB infection and disease signals between complete parenteral immunization (group A) and mucosal immunization (group B) directly. All tests were two-tailed, and results with *p* < 0.05 were considered to be statistically significant. A Spearman *r* correlate analysis was performed of immune response parameters versus disease readouts. The following immune parameters were analyzed: 5-Ag-specific *proliferative* responses (“TCPA PBMC WK34”); 5-Ag-specific *IFN-γ production* (ELISpot); 5-Ag-specific *T cell responses* in the lung (“CD4 BAL WK34” and “CD8 BAL WK34”) and in the blood (“CD4 PBMC WK34” and “CD8 PBMC WK34”) as measured by flow cytometry. The disease readout parameters were total lung pathology (Lung_tot_ PA); bacterial load (“CFU”), and PET signals on WK04 (“WK04 SUV_tot_”), WK08 (“WK08 SUV_tot_”), and WK12 (“WK12 SUV_tot_”).

### Reporting summary

Further information on experimental design is available in the Nature Research Reporting Summary linked to this article.

## Supplementary information

Supplementary Information

Reporting Summary

## Data Availability

All data that support the findings of this study are available from the corresponding authors upon reasonable request.
