# Supplementary material for: Evaluation of heterologous prime-boost vaccination strategies using chimpanzee adenovirus and modified vaccinia virus for TB subunit vaccination in rhesus macaques
Source: NPJ Vaccines. 2020 May 14;5:39. doi: 10.1038/s41541-020-0189-2 (PMC7224290; doi:10.1038/s41541-020-0189-2)
Supplement: Supplementary file 1 — Supplementary Information [file 41541_2020_189_MOESM1_ESM.pdf]

Supplementary Table 1. List of animal's identifiers, gender, date of birth, age and weight at start of the efficacy study per treatment group (including group averages with standard deviation).

Treatment	Stack	Animal ID	Gender	Date of birth (dd-mm-yy)	Age (years)	Starting weight (kg)
A	1	R10028	M	06-05-10	6.5	8.17
	4	R10034	M	11-05-10	6.5	10.03
	2	R11104	M	14-06-11	5.4	9.64
	2	R11105	M	14-06-11	5.4	9.82
	4	R11116	M	22-06-11	5.4	9.80
	1	R11141	M	11-08-11	5.2	9.29
	1	R11143	M	13-08-11	5.2	7.70
	4	R12036	M	30-04-12	4.6	5.99
	3	R12061	M	14-05-12	4.5	7.91
	3	R12145	M	16-07-12	4.3	6.55
average ± sd					5.30 ± 0.75	8.49 ± 1.45
B	4	R10033	M	10-05-10	6.5	10.64
	4	R10041	M	16-05-10	6.5	9.07
	2	R11020	M	24-04-11	5.5	11.74
	3	R11096	M	07-06-11	5.4	7.52
	1	R11111	M	17-06-11	5.4	8.58
	1	R11112	M	17-06-11	5.4	8.99
	2	R12012	M	09-04-12	4.6	8.74
	3	R12072	M	18-05-12	4.5	7.11
	1	R12085	M	24-05-12	4.4	9.73
	4	R12107	M	04-06-12	4.5	7.21
average ± sd					5.27 ± 0.80	8.93 ± 1.48
C	3	R10088	M	13-06-10	6.4	8.41
	3	R10098	M	21-06-10	6.4	8.37
	1	R10101	M	24-06-10	6.4	8.53
	1	R10117	M	05-07-10	6.3	8.36
	4	R11022	M	27-04-11	5.6	8.08
	4	R11098	M	07-06-11	5.5	13.50
	4	R11107	M	15-06-11	5.4	8.96
	1	R11123	M	06-07-11	5.3	8.38
	2	R12051	M	09-05-12	4.5	7.42
	2	R12086	M	25-05-12	4.5	6.54
average ± sd					5.62 ± 0.75	8.66 ± 1.83

Supplementary Table 1. (continued)

Treatment	Stack	Animal ID	Gender	Date of birth (dd-mm-yy)	Age (years)	Starting weight (kg)
D	3	R10001	M	26-03-10	6.6	10.91
	4	R10058	M	01-06-10	6.5	9.38
	2	R11023	M	28-04-11	5.5	9.32
	1	R11028	M	04-05-11	5.5	8.09
	1	R11113	M	17-06-11	5.4	8.46
	4	R11118	M	27-06-11	5.4	9.49
	1	R11136	M	27-07-11	5.3	8.40
	3	R12034	M	28-04-12	4.5	6.46
	4	R12052	M	09-05-12	4.5	6.42
	2	R12095	M	28-05-12	4.4	7.88
average ± sd					5.37 ± 0.75	8.48 ± 1.39
E	2	R09148	M	22-07-09	7.3	12.31
	3	R10014	M	22-04-10	6.6	9.24
	3	R11005	M	07-04-11	5.6	9.91
	4	R11052	M	16-05-11	5.5	9.46
	3	R12018	M	12-04-12	4.6	7.48
	4	R12047	M	06-05-12	4.5	7.29
	1	R12048	M	07-05-12	4.5	7.26
	1	R12057	M	11-05-12	4.5	8.66
	2	R12110	M	04-06-12	4.4	7.87
	2	R12123	M	19-06-12	4.4	8.15
average ± sd					5.19 ± 1.03	8.76 ± 1.56
F	4	R09052	M	11-05-09	8.3	11.25
	3	R10015	M	24-04-10	7.3	10.40
	4	R10090	M	15-06-10	7.2	10.28
	2	R11013	M	17-04-11	6.3	8.77
	1	R11029	M	05-05-11	6.2	7.73
	3	R11041	M	12-05-11	6.2	7.97
	3	R11125	M	09-07-11	6.1	6.59
	2	R12101	M	01-06-12	5.2	8.74
	1	R12108	M	04-06-12	5.1	6.55
	2	R12119	M	15-06-12	5.1	7.40
average ± sd					6.29 ± 1.03	8.57 ± 1.63

Supplementary Table 2. MHC Class I & II genotype of animals in the efficacy study.

Animal and treatment identifiers			MHC Genotype					
			MHC haplotype 1 (parent 1)			MHC haplotype 2 (parent 2)		
Animal ID	Treatment Group	Treatment Abbrev.	A	B	DRB	A	B	DRB
**R10028**	A	$CC^{im}M^{id}$	*A001*	*B012b*	*DRB01a*	*A006*	*B024a*	*DRB06*
**R10034**	A	$CC^{im}M^{id}$	*A001*	*B048*	*DRB15*	*A007*	*B024a*	*DRB11*
**R11104**	A	$CC^{im}M^{id}$	*A006*	*B008*	*DRB14a*	*A008*	*B028*	*DRB09*
**R11105**	A	$CC^{im}M^{id}$	*A012*	*B008*	*DRB10*	*A004*	*B015a*	*DRB04*
**R11116**	A	$CC^{im}M^{id}$	*A002a*	*B012b*	*DRB04*	*A001*	*B048*	*DRB15*
**R11141**	A	$CC^{im}M^{id}$	*A004*	*B015a*	*DRB04*	*A023*	*B012b*	*DRB03f*
**R11143**	A	$CC^{im}M^{id}$	*A004*	*B015a*	*DRB03a*	*A002a*	*B043a*	*DRB01a*
**R12036**	A	$CC^{im}M^{id}$	*A002a*	*B012b/c*	*DRB15*	*A006*	*B008*	*DRB14a*
**R12061**	A	$CC^{im}M^{id}$	*A001*	*B045a*	*DRB10*	*A008*	*B048*	*DRB03a*
**R10033**	B	$CC^{ae}M^{ae}$	A004	B024a	DRB04	A016	B008	DRB04
**R10041**	B	$CC^{ae}M^{ae}$	A001	B055	DRB03f	A008	B001a	DRB03a
**R11020**	B	$CC^{ae}M^{ae}$	A004	B024a	DRB04	A002a	B001a	DRB02
**R11096**	B	$CC^{ae}M^{ae}$	A008	B047a	DRB03e	A004	B024a	DRB04
**R11111**	B	$CC^{ae}M^{ae}$	A004	B024a	DRB04	A004	B012b	DRB06
**R11112**	B	$CC^{ae}M^{ae}$	A008	B001a	DRB03a	A012	B012b	DRB04
**R12012**	B	$CC^{ae}M^{ae}$	A008	B028	DRB09	A002a	B001a	DRB02
**R12072**	B	$CC^{ae}M^{ae}$	A008	B047a	DRB03e	A004	B024a	DRB04
**R12085**	B	$CC^{ae}M^{ae}$	A023	B012b	DRB03f	A004	B048	DRB01a
**R12107**	B	$CC^{ae}M^{ae}$	A006	B008	DRB14a	unknown	B028	DRB09
**R10088**	C	$CC^{im}M^{ae}$	*A023*	*B012b*	*DRB03f*	*A002a*	*B043a*	*DRB01a*
**R10098**	C	$CC^{im}M^{ae}$	*A007*	*B024a*	*DRB11*	*A004*	*B015a*	*DRB04*
**R10101**	C	$CC^{im}M^{ae}$	*A004*	*B012b*	*DRB06*	*A006*	*B024a*	*DRB06*
**R10117**	C	$CC^{im}M^{ae}$	*A004*	*B012b*	*DRB03a*	*A001*	*B048*	*DRB15*
**R11022**	C	$CC^{im}M^{ae}$	*A007*	*B055*	*DRB03F*	*A012*	*B012b*	*DRB04*
**R11098**	C	$CC^{im}M^{ae}$	*A004*	*B024a*	*DRB04*	*A004*	*B001a*	*DRB07*
**R11107**	C	$CC^{im}M^{ae}$	A008	B047a	DRB03e	A004	B024a	DRB04
**R11123**	C	$CC^{im}M^{ae}$	*A004*	*B012b*	*DRB03a*	*A008*	*B028*	*DRB09*
**R12051**	C	$CC^{im}M^{ae}$	*A008*	*B045a*	*DRB05*	*A008*	*B069b*	*DRB08*
**R12086**	C	$CC^{im}M^{ae}$	*A012*	*B012b*	*DRB04*	*A008*	*B069b*	*DRB08*

Supplementary Table 2. (continued)

Animal and treatment identifiers			MHC Genotype					
			MHC haplotype 1 (parent 1)			MHC haplotype 2 (parent 2)		
Animal ID	Treatment Group	Treatment Abbrev.	A	B	DRB	A	B	DRB
**R10001**	D	CC[ae]	A008	B028	DRB09	A008	B001a	DRB03a
**R10058**	D	CC[ae]	A001	B012b/c	DRB06	A008	B001a	DRB03a
**R11023**	D	CC[ae]	A004	B024a	DRB04	A002a	B001a	DRB02
**R11028**	D	CC[ae]	A002a	B043a	DRB01a	A023	B012b	DRB03f
**R11113**	D	CC[ae]	A224a	B045a	DRB01a	A007	B024a	DRB11
**R11118**	D	CC[ae]	A004	B012b	DRB03a	A008	B028	DRB09
**R11136**	D	CC[ae]	A002a	B001a	DRB02	A004	B012c	DRB03f
**R12034**	D	CC[ae]	A002a	B024a	DRB04	A004	B015a	DRB04
**R12052**	D	CC[ae]	A007	B024a	DRB11	A004	B015a	DRB04
**R09148**	E	Non-v	A004	B015a	DRB03a	A002a	B043a	DRB01a
**R10014**	E	Non-v	A026	B012b	DRB01b	A008	B069b	DRB08
**R11005**	E	Non-v	A008	B047a	DRB03e	A002a	B001a	DRB02
**R11052**	E	Non-v	A002a	B001a	DRB02	A002a	B001a	DRB02
**R12018**	E	Non-v	A004	B024a	DRB04	A002a	B001a	DRB02
**R12047**	E	Non-v	A002a	B012b/c	DRB15	A006	B008	DRB14a
**R12048**	E	Non-v	A004	B017a	DRB03a	A008	B001a	DRB03a
**R12057**	E	Non-v	A011	B008	DRB04	A008	B001a	DRB03a
**R12110**	E	Non-v	A002a	B001a	DRB02	A008	B045a	DRB05
**R12123**	E	Non-v	A004	B024a	DRB04	A002a	B001a	DRB02
**R09052**	F	BCG	A004	B001a	DRB01a	A002a	B001a	DRB02
**R10015**	F	BCG	A002a	B001a	DRB02	A008	B069b	DRB08
**R10090**	F	BCG	A002a	B043a	DRB01a	A023	B012b	DRB03f
**R11013**	F	BCG	A004	B017a	DRB03a	A001	B048	DRB03a
**R11029**	F	BCG	A004	B024a	DRB04	A004	B024a	DRB04
**R11041**	F	BCG	A004	B017a	DRB03a	A008	B001a	DRB03a
**R11125**	F	BCG	A001	B055	DRB03f	A001	B048	DRB03a
**R12101**	F	BCG	A008	B045a	DRB05	A004	B024a	DRB04
**R12108**	F	BCG	A026	B047a	DRB03e	A004	B024a	DRB04
**R12119**	F	BCG	A008	B045a	DRB05	A012	B008	DRB10

Supplementary Table 3. List of animal identifiers, gender, date of birth, age and weight at start of the dose ranging study. This study was perormd at Bioqual, Inc. (Rockville, MD, USA)

Dose (vp)	Animal ID	Gender	Date of birth (dd-mm-yy)	Age (years)	Starting weight (kg)
1e9 ChAd3	1006226	F	08-06-10	5,0	4,1
	1002170	F	13-02-10	5,3	4,9
	1006194	F	16-06-11	4,0	5,4
	1005012	F	03-05-11	4,1	5,4
	R588	F	08-04-03	12,2	7,5
1e10 ChAd3	1205200	F	15-05-15	0,1	5,1
	1004228	F	08-04-11	4,2	5,1
	1005186	F	05-05-11	4,1	5,5
	1007204	F	22-07-11	3,9	5,4
	1005182	F	15-05-11	4,1	4,7
1e11 ChAd3	R590	F	10-05-04	11,1	9,1
	1004212	F	23-04-11	4,1	5,2
	1006204	F	24-06-11	4,0	4,7
	1004224	F	25-04-11	4,1	5,1
	5266	F	21-05-06	9,1	8,7

Supplementary Table 4.1. Macroscopic TB disease manifestation is arbitrarily scoring the number, nature and size of lesions, and is applied for lung lobes (individually), extrathoracic/abdominal vicera as well as other thoracic/non-pulmonary lesions. The maximally possible score is 10 in case of extensive consolidation and coalescing lesions. Adapted from [1, 2]

Number of Granulomas	Score
no visible lesions	0
1 to 3 lesions	1
4 to 10 lesions	2
10 to 20 lesions	3
20 to 50 lesions	4
> 50 lesions, multifocal areas	5
Extensive Consolidation / TB pneumonia	6
Miliary pattern	7
**Size of Granulomas**	
none	0
< 2 mm	1
2 to 4 mm	2
> 4 mm	3
coalescing lesions	4

Supplementary Table 4.2. Pathological lymph node (LN) involvement is scored by macroscopic manifestation and lymph node enlargement. Lung-draining hilar LN scores are scored on average for 4 distincitve regions: left versus right and cranial versus subcarinal (above and beneath the bifurcation of the trachea, respectively), while inguinal and axial LNs are scored on average for left versus right, respectively.

Gross Manifestation (at cut surface)	Score
no involvement (or not assessable)	0
(multi-)focal, circumscribed, non-coalescing, < 2 mm	1
coalescing solid or caseating, <50% of nodal architecture	2
coalescing solid or caseating, >50% of nodal architecture	3
complete nodal effacement and caseation	4
Size of Lymph Node	
not assessable / normal (< 5 mm)	0
mildly enlarged (5-10 mm)	1
markedly enlarged (>10 mm)	2

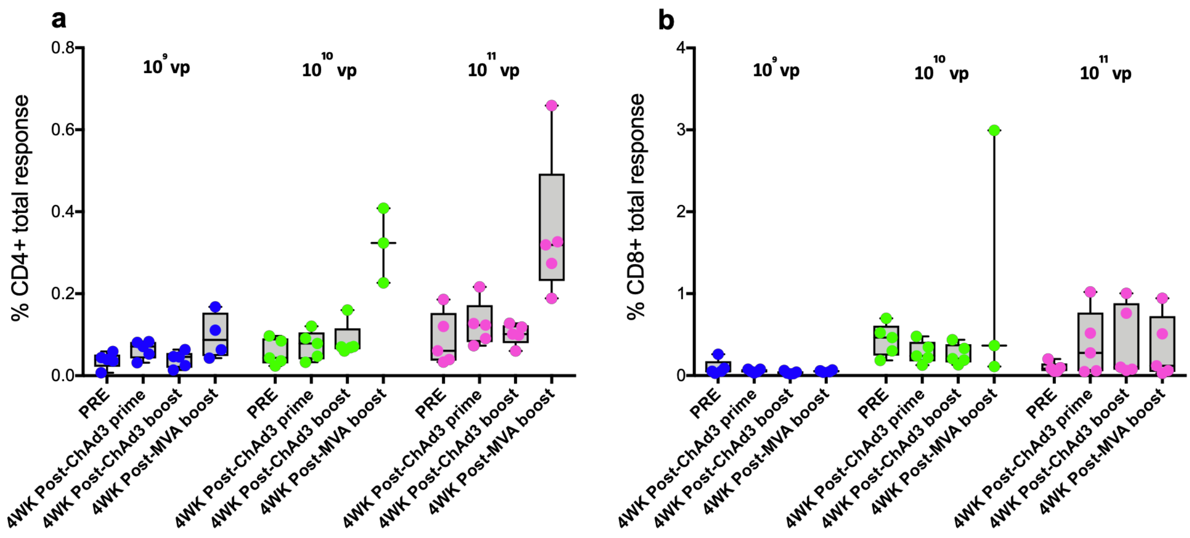

Supplementary Fig. 1. Antigen specific CD4+ and CD8+ T cell in blood measured for different ChAd3 vaccine doses. **a** Percentage of CD4+ or **b** CD8+ T cells from PBMC, producing either IFN-γ, TNF-α and/or IL-2 (in total) after antigen-specific *ex vivo* stimulation with peptide pools of individual antigens contained in the respective viral vector constructs. PBMC samples were collected before vaccination (PRE), 4 weeks after ChAd3-5Ag priming (study week WK4), ChAd3-5Ag boosting (study week WK14) and MVATG18633 (10^8 PFU) boosting (study week Wk28). vp = viral particles. The box extends from the 25th to 75th percentiles. The line in the middle signifies the median and the whiskers include the smallest value up to the largest.

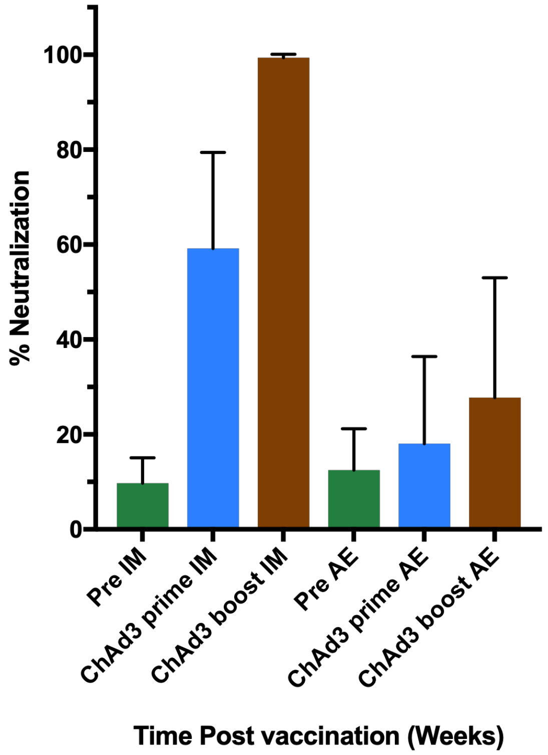

Supplementary Fig. 2. Development of neutralizing antibodies against the ChAd3 vaccine vector**.** The mean neutralization (± stdev) afer parenteral vaccination (IM; Group A & C; N=20) and mucosal vaccination (AE vaccination; Group B & D; N=20) is depicted. Serum samples were diluted 10-fold and tested for the presence of neutralizing antibodies against ChAd3-GFP 4 weeks after the first and second vaccination.

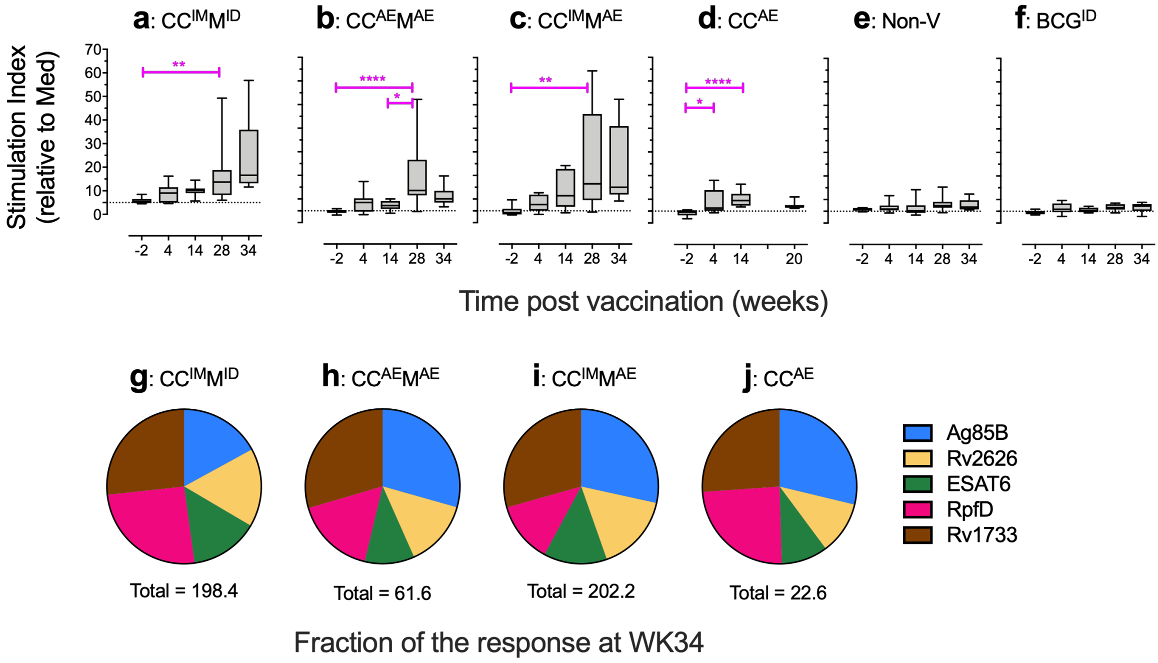

Supplementary Fig. 3. Ag specific lymphocyte proliferation along the vaccination phase as measured by ^{3}H-Thymidine incorporation**.** (**a-f**) Longitudinal display of the sum of Ag specific proliferation against the 5 individual TB antigens measured by ^{3}H-Thymidine incorporation and expressed as stimulation index over the values obtained by culture medium control stimulation. The proliferation assay was performed prior to (WK-2), 4 weeks after ChAd3 priming (WK04), 2 weeks after ChAd3 boosting (WK14), 2 weeks after MVA-5Ag boosting (WK28), and 4 weeks before infectious challenge with *Mtb* (WK34). The box extends from the 25th to 75th percentiles. The line in the middle signifies the median and the whiskers include the smallest value up to the largest.

Statistical significance (by Kruskal-Wallis with Dunn's correction for multiple analysis) relative to pre-vaccination time point (WK-2) is indicated as follows: $p < 0.05$ by *; $p < 0.01$ by **; $p < 0.001$ by ***; $p < 0.0001$ by ****.

(**g-j**) The fraction of the total Ag-specific proliferative response for each individual antigen for each experimental treatment group 4 weeks before challenge (WK34) are displayed. The "Total" number indicated below the pie charts represent the sum of stimulation indices for the 5 TB Ag.

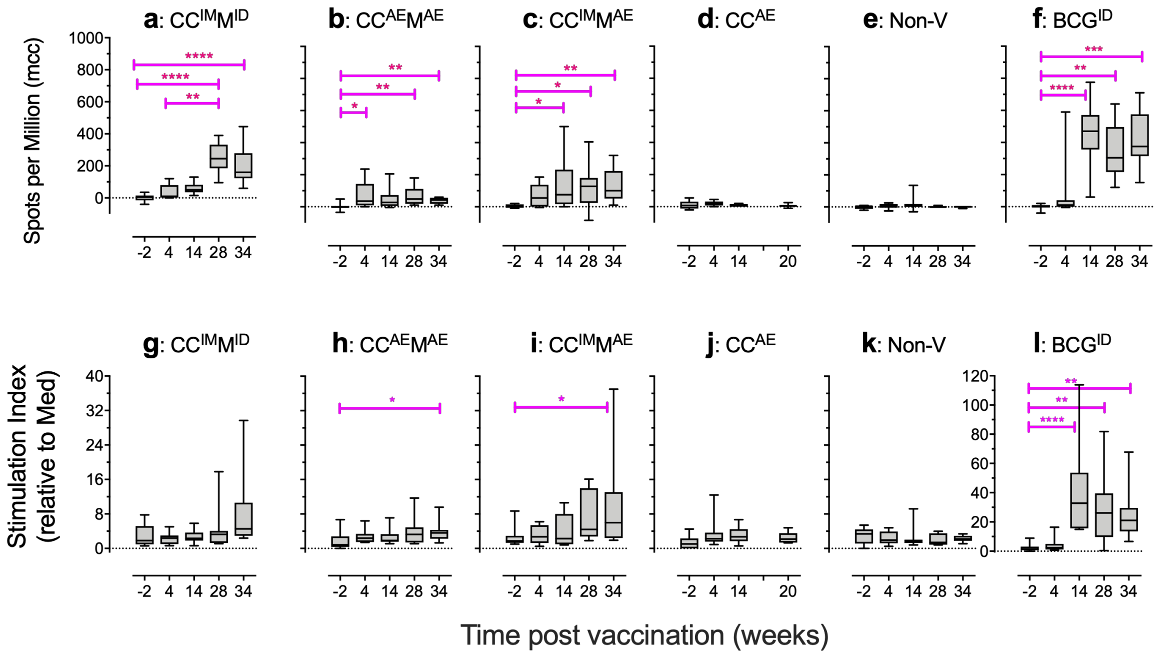

Supplementary Fig. 4. PPD specific IFN-γ secretion and lymphocyte proliferation along the vaccination phase. (**a-f**) Longitudinal display per treatment group (treatment group A to F) of PPD-specific IFN-γ production, measured by ELISpot and expressed as spots per million cells after correction for culture medium control values. IFN-γ ELISPOT was performed prior to immunisation (WK-2), 4 weeks after ChAd3 priming (WK04), 4 weeks after ChAd3 boosting (WK14), 2 weeks after MVA-5Ag boosting (WK28), and 4 weeks before infectious challenge with *Mtb* (WK34).

(**g-l**) Longitudinal display per treatment group (group A to F) of PPD-specific proliferation, measured by ^{3}H-Thymidine incorporation and expressed as stimulation index over the values obtained by culture medium control stimulation. The proliferation assay was performed prior to immunisation (WK-2), 4 weeks after ChAd3 priming (WK04), 2 weeks after ChAd3 boosting (WK12), 2 weeks after MVA-5Ag boosting (WK28), and 4 weeks before infectious challenge with *Mtb* (WK34). The box extends from the 25th to 75th percentiles. The line in the middle signifies the median and the whiskers include the smallest value up to the largest. Statistical significance (by Kruskal-Wallis with Dunn's correction for multiple analysis) relative to pre-vaccination time point (WK-2) is indicated as follows: $p < 0.05$ by *; $p < 0.01$ by **; $p < 0.001$ by ***; $p < 0.0001$ by ****.

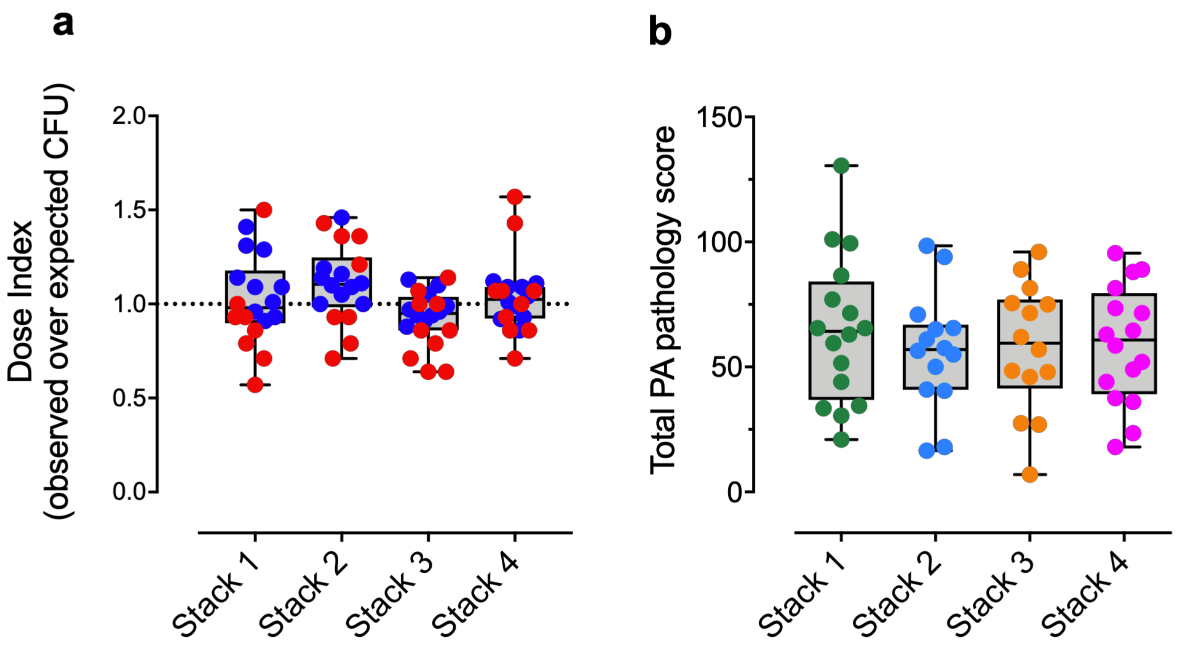

Supplementary Fig. 5. Quality control of inoculum with Mtb Erdman K01 per stack**. a** Serial dilutions from stock were plated in 10 replicates. Dilutions in the countable range (dilution 3 (*bleu*) and dilution 4 (*red*) were used to calculate the index of observed CFU/ expected CFU. Dilution 4 is the dilution prior to the final inoculum. **b** Pathology scores per stack, showing a similar distribution of total gross pathology scores that is not affected by the independent inoculum preparations. The box extends from the 25th to 75th percentiles. The line in the middle signifies the median and the whiskers include the smallest value up to the largest.

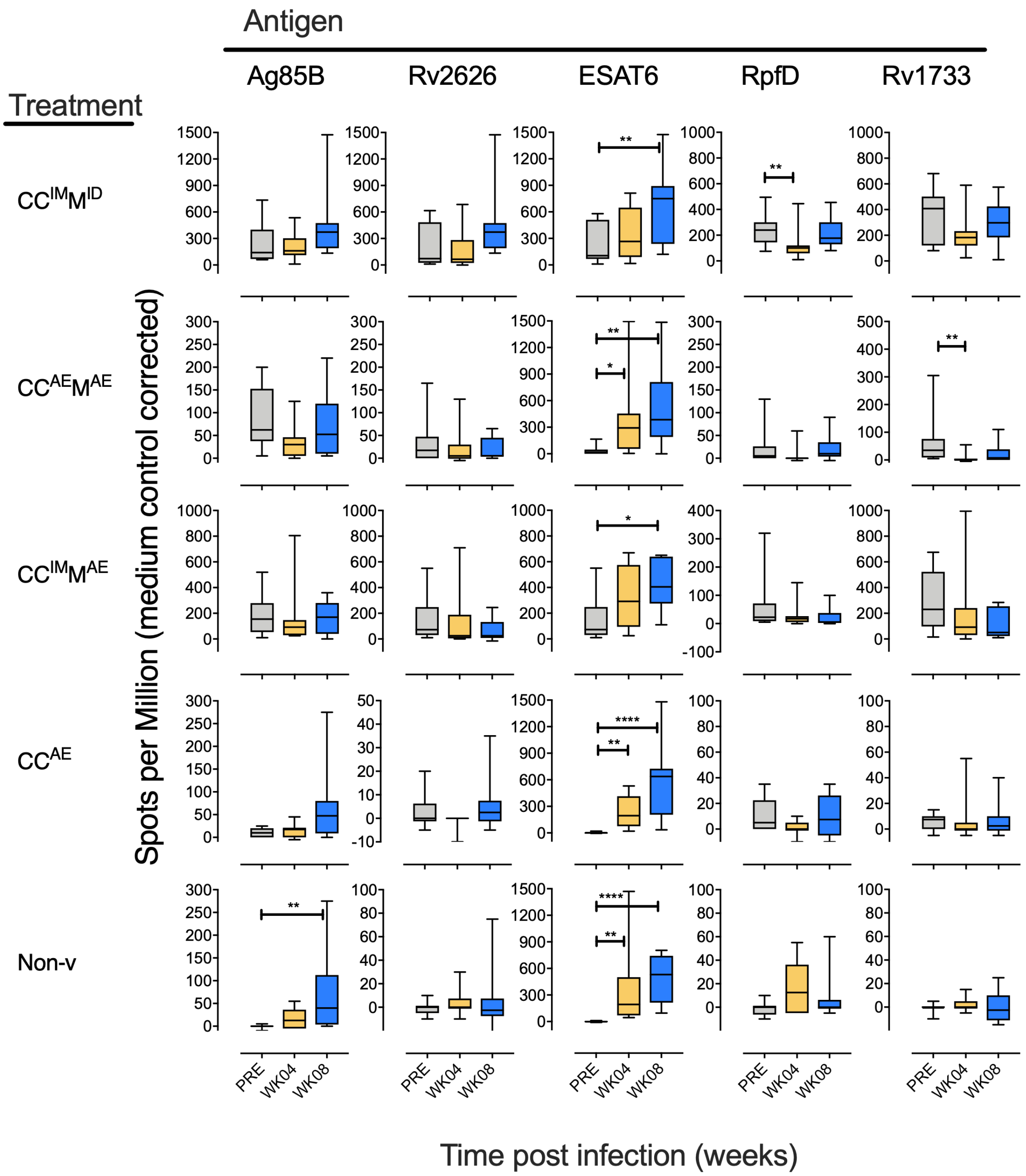

Supplementary Fig. 6. Analysis of anamnestic responses by ELISpot per individual vaccine antigen. Ag specific IFN-γ production 4 weeks before infection (PRE) and 4 and 8 weeks post infection. The different treatment arms are indicated on the left. Statistical significance (by Kruskal-Wallis with Dunn's correction for multiple analysis) relative to pre-vaccination time point (PRE) is indicated as follows: $p < 0.05$ by *; $p < 0.01$ by **; $p < 0.001$ by ***; $p < 0.0001$ by ****.

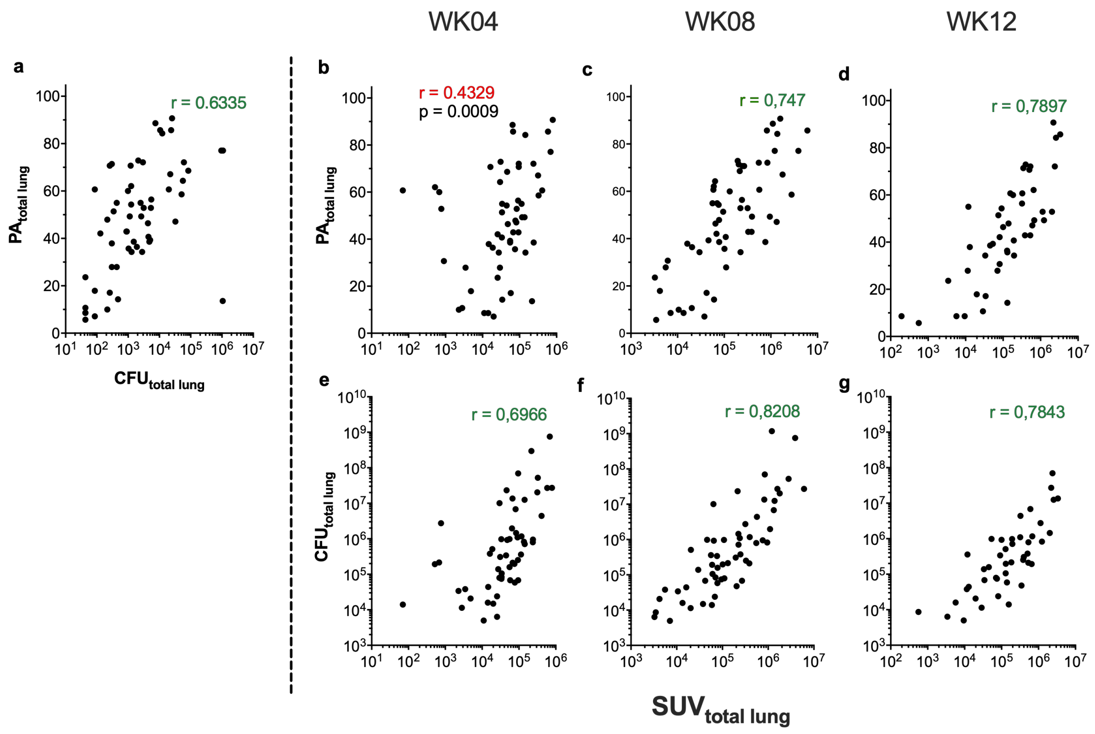

Supplementary Fig. 7. Correlation analysis of major pulmonary TB disease measures. Non-parametric Spearman's rho statistics was applied to assess the correlation between three major readout parameters of efficacy: total lung pathology score (lung PA), mycobacterial burden in the total lung (lung CFU), and total lung standard uptake value (SUV) of ^{18}F-FDG PET-CT (total SUV) 4, 8 and 12 weeks after infectious challenge. For all comparisons, strong (0.6 < r < 0.8) to very strong (0.8 < r < 1) correlations were found (p<0,0001; except for **b**). (**A**) lung PA versus lung CFU, (**B**) lung PA versus SUV at WK4, (**C**) lung PA versus SUV at WK8, (**D**) lung PA versus SUV at WK12, (**E**) lung CFU versus SUV at WK4, (**F**) lung CFU versus SUV at WK8 and (**G**) lung CFU versus SUV at WK12.

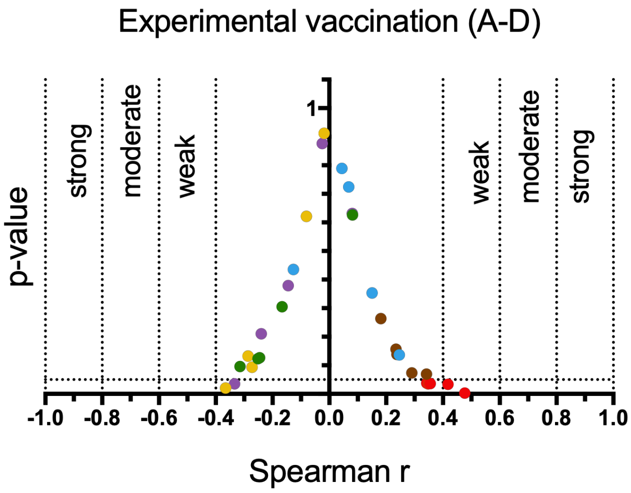

Supplementary Fig. 8. Correlation of immune responses parameters (at week 34) versus disease readout parameters. the Spearman r and corresponding p-value are depicted. **A**) The immune parameters that are color coded for vaccine antigen (5-Ag) specific responses are: *proliferation* (purple); *IFN-γ production* (orange; ELIspot); CD4 *T cell cytokine responses* in the lung (red); CD8 *T cell cytokine responses* in the lung (brown); CD4 *T cell cytokine responses* in the blood (blue); CD8 *T cell cytokine responses* in the blood (green). The disease readout parameters are total lung pathology, CFU and PET signals on week 4, 8 and 12. The strength of correlation is also indicated at the top of the graph from weak over moderate to strong. A signficance threshold p-value of 0.05 is indicated by the horizontal dashed line. The box extends from the 25th to 75th percentiles. The line in the middle signifies the median and the whiskers include the smallest value up to the largest.

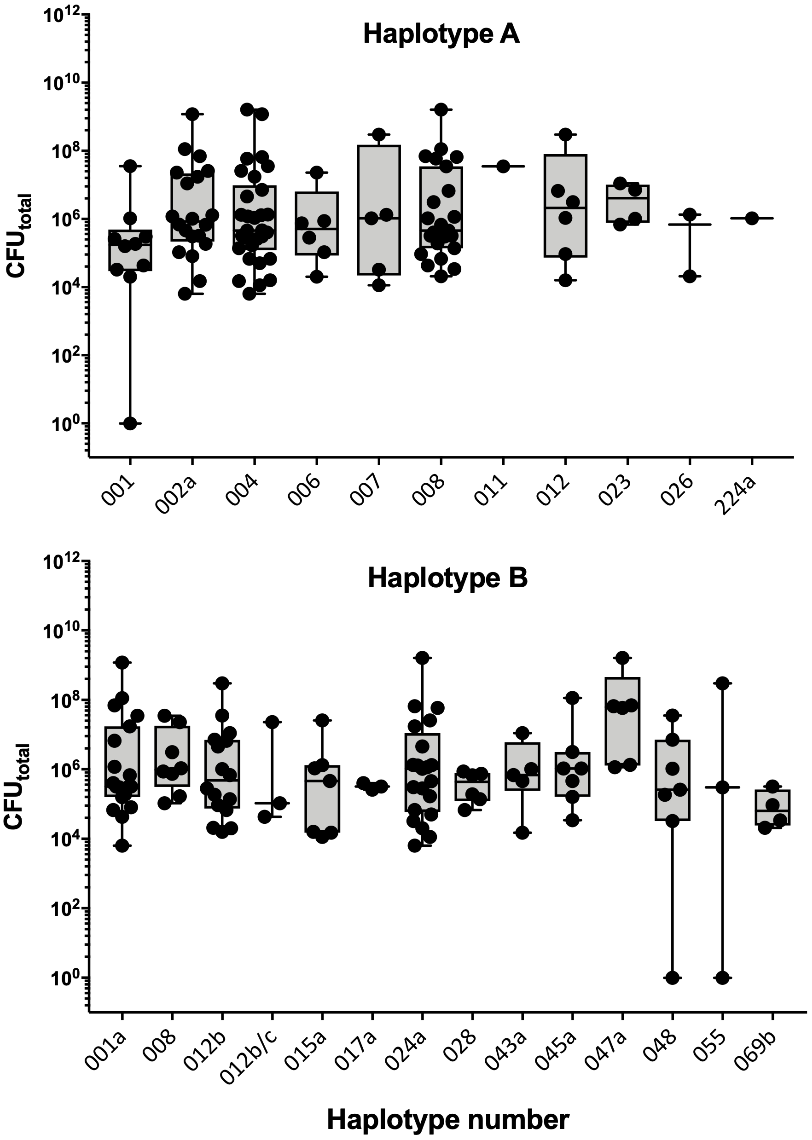

Supplementary Fig. 9. Analysis of bacterial load in the lung for MHC Class I haplotypes (Haplotype A and Haplotype B) for animals of the efficacy experiment (listed in Supplementary Table 2B). Performing ANOVA statistics demonstrated that there was no significant difference between any of these groups. The box extends from the 25th to 75th percentiles. The line in the middle signifies the median and the whiskers include the smallest value up to the largest.

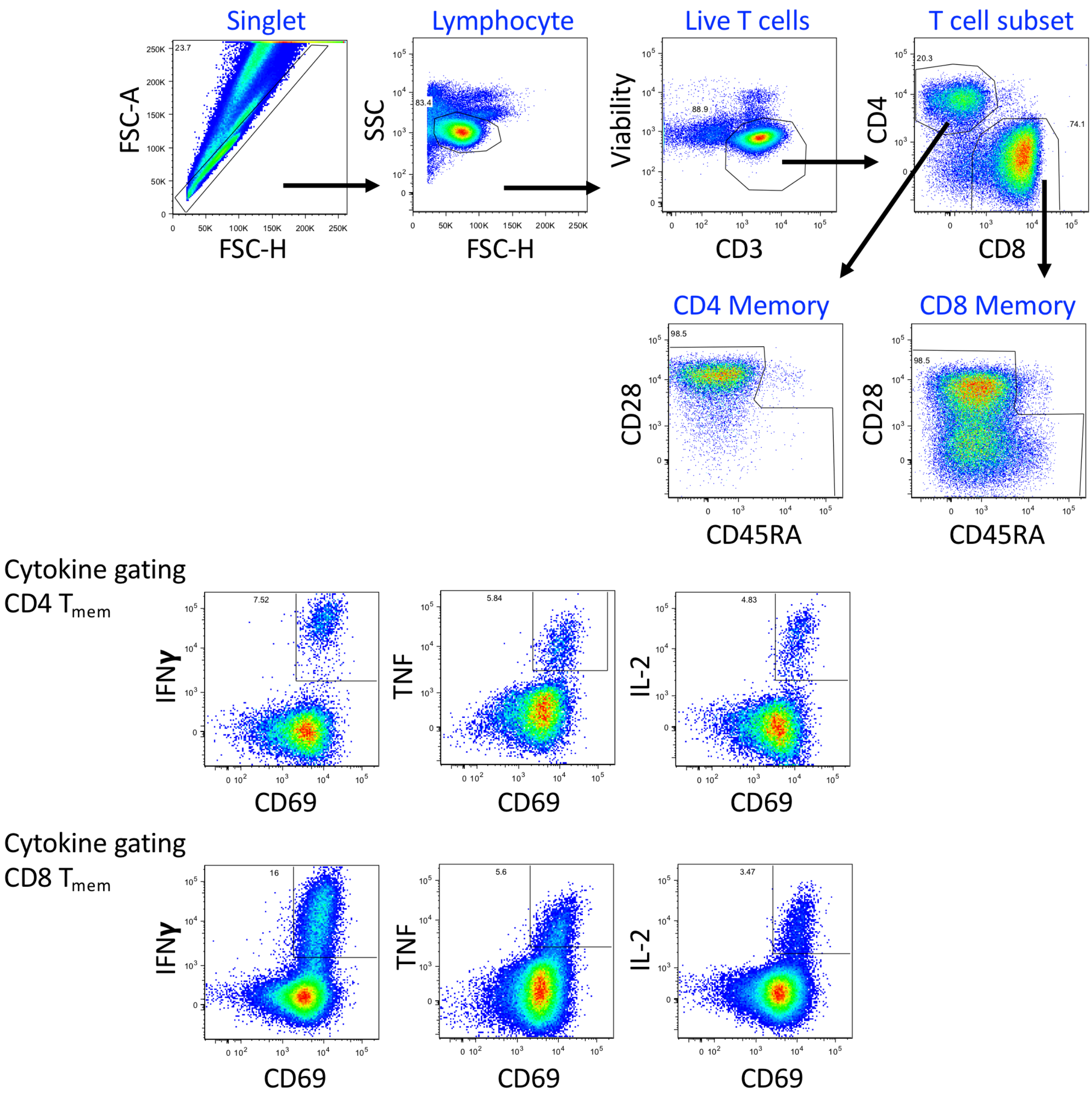

Supplementary Fig. 10. Gating strategy dose-ranging study

Fresh PBMC samples unstimulated (medium control) and PPD stimulated. 1) Gating on singlets; 2) Gating on lymphocytes (FSC/ SSC) 3) Gating on live cells (L/D stain) versus CD3^{+ve} cells; 4) Gating on CD3^{+ve} cells and subsequent gating on CD4 versus CD8; 5) Gating on CD28 versus CD45RA to select for CD4 and CD8 memory T cells; 6) Subsequent gating on CD69 versus cytokines (IL-2/IFN-γ/TNF-α).

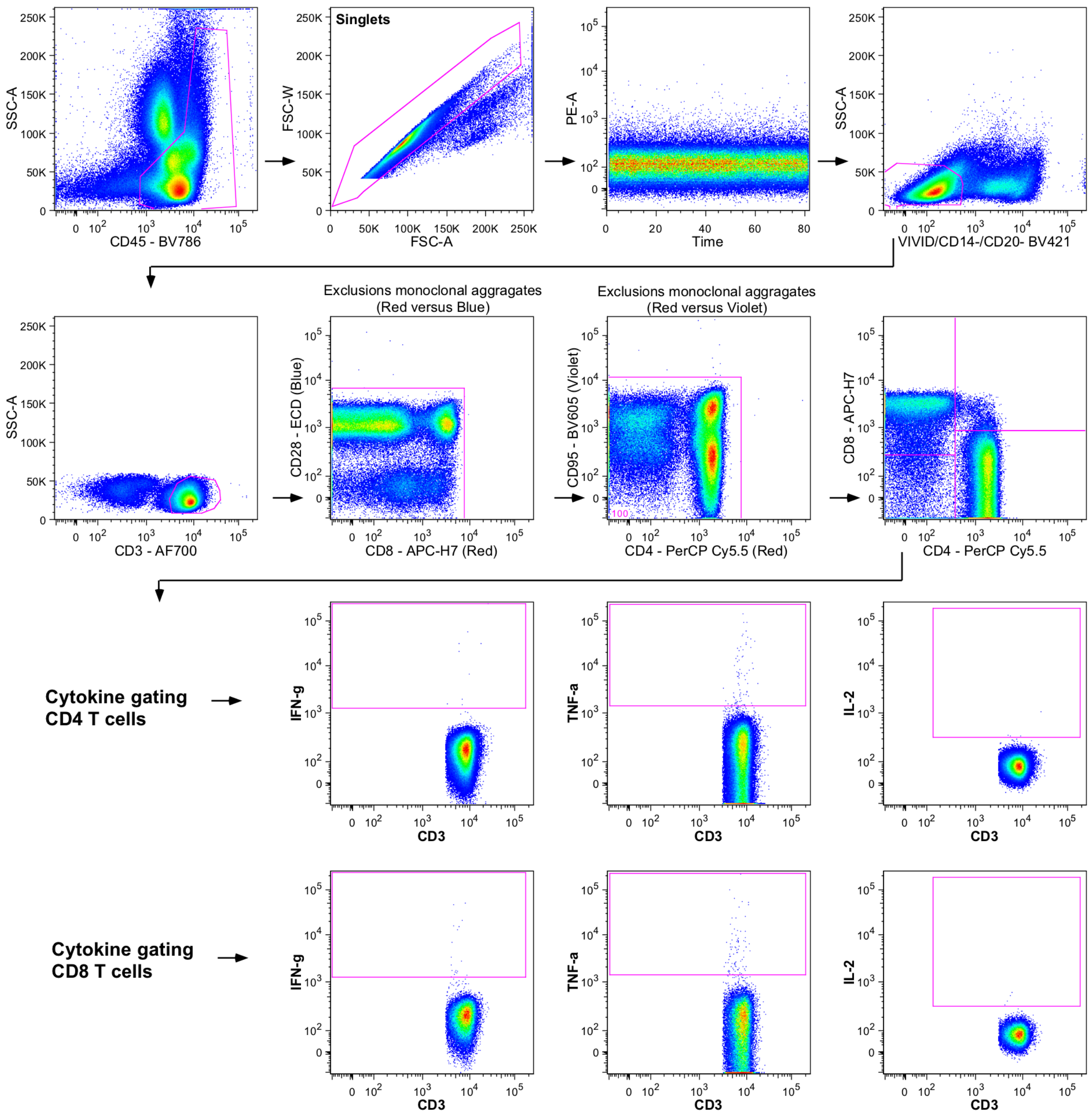

Supplementary Fig. 11. Gating strategy efficacy study.

Fresh PBMC samples unstimulated (medium control) and PPD stimulated. 1) Gating on CD45^{+ve} cells (including all leukocytes)/ SSC); 2) Gating on singlets; 3) Gating out death cells (L/D stain) and CD20^{+ve}/CD14^{+ve} cells (excluding B cells and monocytes in one channel) versus CD3^{+ve} cells (including all T cells); 4) Gating on CD3^{+ve} cells and subsequent gating on CD4 versus CD8. 5) Boolean gating of any cytokine expression of IL-2/IFN-γ/TNF-α.

References

1. Lin PL, Coleman T, Carney JP, Lopresti BJ, Tomko J, Fillmore D, Dartois V, Scanga C, Frye LJ, Janssen C *et al*: **Radiologic responses in cynomolgous macaques for assessing tuberculosis chemotherapy regimens**. *Antimicrob Agents Chemother* 2013.
2. Lin PL, Rodgers M, Smith L, Bigbee M, Myers A, Bigbee C, Chiosea I, Capuano SV, Fuhrman C, Klein E *et al*: **Quantitative comparison of active and latent tuberculosis in the cynomolgus macaque model**. *Infect Immun* 2009, **77**(10):4631-4642.
